# Early vs Delayed Antihypertensive Treatment in Acute Single Subcortical Infarction: A Secondary Analysis of the CATIS-2 Randomized Clinical Trial

**DOI:** 10.1001/jamanetworkopen.2024.30820

**Published:** 2024-08-30

**Authors:** Yufei Wei, Xuewei Xie, Yuesong Pan, Mengxing Wang, Aili Wang, Dacheng Liu, Zilin Zhao, Ximing Nie, Wanying Duan, Xin Liu, Zhe Zhang, Jingyi Liu, Lina Zheng, Suwen Shen, Chongke Zhong, Tan Xu, Yong Jiang, Jing Jing, Xia Meng, Katherine Obst, Chung-Shiuan Chen, Hao Li, Xinyi Leng, David Wang, Yilong Wang, Yonghong Zhang, Jiang He, Yongjun Wang, Liping Liu

**Affiliations:** 1 Department of Neurology, Beijing Tiantan Hospital, Capital Medical University, Beijing, China; 2 China National Clinical Research Center for Neurological Diseases, Beijing, China; 3 Department of Epidemiology, School of Public Health, Suzhou Medical College of Soochow University, Suzhou, China; 4 Department of Neurology, The Second Affiliated Hospital of Xi’an Jiaotong University, Xi’an, China; 5 Department of Medical Administration, Suzhou Industrial Park Medical and Health Management Center, Suzhou, China; 6 Department of Epidemiology, Tulane University School of Public Health and Tropical Medicine, New Orleans, Louisiana; 7 Tulane University Translational Science Institute, New Orleans, Louisiana; 8 Department of Medicine and Therapeutics, Chinese University of Hong Kong, Hong Kong SAR, China; 9 Neurovascular Division, Department of Neurology, Barrow Neurological Institute, St Joseph’s Hospital and Medical Center, Phoenix, Arizona; 10 Advanced Innovation Center for Human Brain Protection, Beijing Tiantan Hospital, Capital Medical University, Beijing, China; 11 Research Unit of Artificial Intelligence in Cerebrovascular Disease, Chinese Academy of Medical Sciences, Beijing, China

## Abstract

**Importance:**

The China Antihypertensive Trial in Acute Ischemic Stroke II (CATIS-2) suggests that early antihypertensive treatment did not reduce the risk of dependency or death in acute ischemic stroke (AIS), compared with delayed treatment. Single subcortical infarction (SSI) is an important stroke subtype, and the association of antihypertensive timing with clinical outcomes is unclear.

**Objective:**

To investigate the association of early vs delayed antihypertensive treatment with clinical outcomes in patients with SSI, stratified by the presence of parent artery disease (PAD) stenosis.

**Design, Setting, and Participants:**

This secondary analysis of the CATIS-2 randomized clinical trial included 106 hospitals in China between June 2018 and July 2022. In CATIS-2, patients with AIS within 24 to 48 hours of symptoms onset and elevated systolic blood pressure were eligible. Patients with SSI detected in diffusion-weighted imaging were included in the current post hoc subgroup analysis. Patients were grouped into (1) SSI with PAD stenosis and (2) SSI without PAD stenosis. Statistical analysis was performed from July 2023 to May 2024.

**Exposures:**

Early (immediate) vs delayed (starting on day 8) antihypertensive therapy.

**Main Outcome and Measure:**

Primary outcome was the combination of functional dependency or death (modified Rankin Scale score ≥3) at 90 days.

**Results:**

Among 997 patients with SSI in CATIS-2 (mean [SD] age, 62.4 [9.8] years; 612 [61.4%] men), 116 (11.6%) had SSI with PAD and 881 (88.4%) had SSI without PAD. There was no significant difference in the primary outcome between early and delayed antihypertensive treatment groups among all patients with SSI (8.8% vs 7.1%; OR, 1.25 [95% CI, 0.79-1.99]; *P* = .34). Among patients with SSI with PAD, early antihypertensive treatment was associated with increased risk of the primary outcome compared with delayed treatment (23.4% vs 7.7%; OR, 3.67 [95% CI, 1.14-11.86]; *P* = .03); this finding was not observed in patients with SSI without PAD (6.6% vs 7.1%; OR, 0.93 [95% CI, 0.55-1.57]; *P* = .77). Significant interaction with treatment and presence of PAD stenosis was detected for the primary outcome (*P* for interaction = .04).

**Conclusions and Relevance:**

In this secondary analysis of a randomized clinical trial, early antihypertensive treatment was associated with an increased risk of functional dependency or death at 90 days among patients with SSI and coexisting PAD stenosis, compared with delayed antihypertensive treatment. Further studies are warranted for individualized BP management in patients with SSI by the presence of PAD.

**Trial Registration:**

ClinicalTrials.gov Identifier: NCT03479554

## Key Points

**Question:**

Is there an association of antihypertensive treatment timing with clinical outcomes among patients with acute ischemic stroke with single subcortical infarction, and does the association differ depending on the presence of parent artery disease stenosis?

**Findings:**

In this secondary analysis of a randomized clinical trial that included 997 patients with acute single subcortical infarction and elevated blood pressure within 24 to 48 hours after stroke onset, early antihypertensive treatment was associated with an increased risk of functional dependency or death at 90 days among those with coexisting parent artery disease stenosis, compared with delayed antihypertensive treatment.

**Meaning:**

These results suggest that early blood pressure reduction should be approached with caution in patients with acute single subcortical infarction with parent artery disease stenosis.

## Teaser

This secondary analysis examines whether there is an association of early vs delayed antihypertensive treatment with outcomes among Chinese patients with single subcortical infarction, stratified by the presence of parent artery disease stenosis.

## Introduction

Single subcortical infarction (SSI) is typically characterized as a single subcortical lesion within the territory of perforating arteries, accounting for 20% to 30% of all ischemic strokes.^1,2,3^ Elevated blood pressure (BP) is more obvious in the acute phase among patients with SSI compared with other stroke subtypes and is associated with adverse prognosis.^4,5,6^ Therefore, BP management of this important stroke subtype is worth exploring.

There have been studies investigating the optimal target of BP reduction in patients with SSI.^7,8^ Although, the timing of initiating antihypertensive treatment was not considered in investigating the associations between BP levels and outcomes of patients with SSI in these studies. A recent registry study suggested different relationships between systolic BP (SBP) levels measured at different time points after stroke and the functional outcomes in patients with SSI.^9^ However, with limited evidence on the effects of antihypertensive treatments initiating at different time points after stroke on outcomes of patients with SSI, there is no consensus on the optimal timing of initiating antihypertensive treatment in such patients.

SSI was often considered as a lacunar stroke or cryptogenic stroke previously,^2,3,10,11^ while emerging evidence has suggested different etiologies of SSI. Either lipohyalinosis and fibrinoid degeneration of perforators or parent artery atherosclerosis blocking the orifice of perforators could cause SSI.^3,10,12,13,14,15,16^ SSI with coexisting parent artery disease (PAD) are common in Asian populations, among whom intracranial artery stenosis is prevalent.^17,18^ Nevertheless, it is unknown whether presence of PAD stenosis would affect the association between BP management strategy and outcomes of patients with SSI.

China Antihypertensive Trial in Acute Ischemic Stroke-2 (CATIS-2), a multicenter randomized clinical trial, found that early antihypertensive treatment starting within the first 24 to 48 hours did not reduce the odds of dependency or death at 90 days among patients with acute ischemic stroke (AIS), compared with delayed treatment starting on day 8 after randomization.^19^ We conducted a subgroup analysis of the CATIS-2 trial to investigate the association of early vs delayed antihypertensive treatment with clinical outcomes in patients with acute SSI, and to evaluate such associations by the presence of PAD stenosis.

## Methods

### Study Design and Participants

The CATIS-2 trial is a multicenter, randomized, open-label, blinded–end point trial conducted in 106 hospitals across China from June 2018 through July 2022, which aimed to investigate the effect of early antihypertensive treatment vs delayed treatment on reducing functional dependency or death in patients with AIS (trial protocol in Supplement 1 and statistical analysis plan in Supplement 2). Details on the rationale, design, and overall findings of the CATIS-2 trial have been published.^19,20^ Briefly, patients aged at least 40 years with AIS within 24 to 48 hours of symptom onset and SBP between 140 and 220 mm Hg were enrolled. The major exclusion criteria included atrial fibrillation and receipt of intravenous thrombolytic treatment or endovascular thrombectomy at baseline. Participants were randomly assigned in 1:1 ratio to receive antihypertensive treatment immediately after randomization (aimed at reducing SBP by 10% to 20% within the first 24 hours and achieving mean BP <140/90 mm Hg within 7 days) or to discontinue antihypertension medications for 7 days and then restart treatment on day 8 (aimed at achieving mean BP <140/90 mm Hg). This trial was approved by the ethics committee of Beijing Tiantan Hospital and all participating institutes in China, and by the institutional review board of Tulane University in the US. Written informed consent was obtained from patients or their representatives. We followed the Consolidated Standards of Reporting Trials (CONSORT) reporting guideline.

The current study is a post hoc subgroup analysis of the CATIS-2 trial. Patients in the CATIS-2 trial who met the following criteria were included in the current study: (1) having a brain magnetic resonance imaging (MRI) examination (1.5- or 3.0-T) during hospitalization, with sequences including diffusion-weighted imaging (DWI) and 3D time-of-flight (TOF) magnetic resonance angiography (MRA); (2) having an SSI in the perforator territory of middle cerebral artery (MCA), basilar artery (BA) or posterior cerebral artery (PCA). Patients (1) with poor image quality or without DWI or TOF-MRA assessment, (2) with potential cardiac embolic sources (eg, newly diagnosed atrial fibrillation, rheumatic heart disease, or recent myocardial infarction), or (3) with at least 50% stenosis of ipsilateral extracranial large artery, were excluded as in previous studies.^21,22,23,24,25^

### Data Collection

The demographic characteristics, medical history, and previous medication use were collected by trained research coordinators at enrollment. Stroke severity was assessed using the National Institutes of Health Stroke Scale (NIHSS) by trained neurologists at baseline, 14-day or hospital discharge, and 90-day follow-up visits. BP measurements were conducted by trained nurses using an automated device (Omron HBP-1300 Professional BP Monitor) according to a standard protocol recommended by the American Heart Association.^26^ Three BP measurements were obtained at baseline; every 3 hours after randomization for the first 24 hours; every 8 hours from day 2 until day 14 or hospital discharge; and at 21-day and 90-day follow-up visits.

### Imaging Assessment

Brain MRI scans among patients enrolled in the trial were completed based on clinical judgement at the individual centers. Such decisions were made independent of treatment allocation and were done primarily to assess the infarct and intracranial vessels. And imaging data were collected from individual centers in Digital Imaging and Communications in Medicine format. Centralized interpretation of the imaging data was independently conducted by 2 experienced neurologists blinded to clinical data. A third neuroradiologist was involved for additional assessment if there was disagreement in certain cases. SSI was defined as a single DWI lesion in the perforator territory of MCA, BA, and PCA, such as the striatocapsular, thalamic, or brainstem area. Relevant PAD stenosis was defined as presence of 50% to 99% stenosis or occlusion of the parent artery in TOF-MRA that corresponds to SSI lesions (eg, MCA [M1 segment], BA, and PCA [P1/P2 segment]). Patients with SSI included in this study were categorized into 2 groups based on presence of PAD stenosis (eFigure 1 in Supplement 3): (1) SSI with PAD stenosis and (2) SSI without PAD stenosis.

### Outcomes Measurements

The primary outcome of the current study was the combination of functional dependency or death (modified Rankin Scale [mRS] score ≥3) at 90 days after randomization. Secondary outcomes included ordinal mRS scores, recurrent stroke, and major vascular disease events (ie, vascular deaths, nonfatal stroke, nonfatal myocardial infarction, coronary revascularization, and hospitalized angina and heart failure) at 90 days, as well as early neurological deterioration (END; an increase of ≥2 in the total NIHSS score during hospitalization). The outcome assessments were conducted on site through follow-up visits by trained research nurses and neurologists, and all primary and secondary outcomes were reviewed by an Outcome Adjudication Committee, with all involved study staff blinded to the treatment assignment.

### Statistical Analysis

Data were analyzed using modified intension-to-treat analysis excluding certain participants who withdrew consent. Baseline characteristics were presented as mean (SD) or median (IQR) for continuous variables and numbers (percentages) for categorical data. Linear mixed-effects regression analysis was used to test the group differences in mean BP changes between the early and delayed treatment groups with a significance level of .004 (.05 / 14 tests). Logistic regression analysis was used to estimate odds ratios (ORs) and 95% CIs associated with early treatment compared with delayed treatment. In addition, ordinal logistic regression was used to estimate the effect of early antihypertensive treatment compared with delayed treatment on the full range of the mRS scores. Complete case analysis was used for the primary analyses. Multiple imputation was conducted using the Markov chain Monte Carlo method to handle missing primary outcome data in a sensitivity analysis. We assessed the heterogeneity of the treatment association with primary and secondary outcomes according to the PAD stenosis status by adding an interaction term in the logistic regression models. Sensitivity analysis was conducted to verify the stability of the model by adjusting for potential confounders including age, sex, baseline NIHSS score, history of hypertension, diabetes, dyslipidemia and stroke, previous medication use of antihypertensive, antiplatelet and lipid-lowering therapy, and time from onset to imaging. Statistical significance was defined by 2-sided *P* < .05. All statistical analyses were performed using SAS software version 9.4 (SAS Institute) from July 2023 to May 2024.

## Results

### Baseline Characteristics

Among 4810 patients with AIS with elevated BP enrolled in CATIS-2, 2897 patients underwent brain 1.5- or 3.0-T magnetic resonance examination during hospitalization. After excluding 69 patients without DWI, 450 without MRA, and 7 with poor image quality, the infarct pattern and presence of intracranial artery stenosis were assessed in 2389 patients. Furthermore, 1364 patients without SSI, 5 with potential cardiac embolic sources, and 23 with at least 50% stenosis of ipsilateral extracranial large artery were excluded. Finally, a total of 997 patients with SSI were included in the current analyses (eFigure 2 in Supplement 3). Baseline characteristics between CATIS-2 patients with and without MRI scans during hospitalization were compared (eTable 1 in Supplement 3). Compared with those without MRI scans (n = 1905), patients with MRI scans were slightly younger, had higher baseline SBP and proportion of previous antiplatelet and lipid-lowering therapy use, more likely to be current smokers and categorized as having small artery disease, and had longer time from stroke onset to randomization.

Among the 997 included patients, 116 (11.6%) had SSI with PAD (of which 13 cases had PAD occlusion) and 881 (88.4%) had SSI without PAD; 612 patients (61.4%) were men; and the mean (SD) age was 62.4 (9.8) years. The baseline characteristics between the 2 treatment groups were well balanced except for a higher proportion of previous antihypertensive therapy use in the delayed treatment group (eTable 2 in Supplement 3). The median (IQR) diameter of infarction was 12.5 (9.8-16.6) mm for patients with SSI with PAD and 11.0 (8.0-15.0) mm for patients with SSI without PAD. Compared with patients with SSI without PAD, patients with SSI with PAD were older, less likely to be male and current smokers, and had higher baseline SBP (Table 1). Baseline characteristics were mostly balanced between the early and delayed antihypertensive treatment respectively in the SSI with PAD and SSI without PAD groups except for history of hypertension, history of diabetes, previous stroke, and previous antihypertensive therapy use (Table 1).

**Table 1.** Baseline Characteristics of the Included Patients With SSI With vs Without PAD

Characteristic	Patients with SSI, No. (%)					
	With PAD			Without PAD		
	Total (N = 116)	Early treatment (n = 64)	Delayed treatment (n = 52)	Total (N = 881)	Early treatment (n = 427)	Delayed treatment (n = 454)
Age, mean (SD), y	65.3 (10.2)	66.4 (10.4)	63.9 (9.8)	62.0 (9.7)	62.2 (9.6)	61.8 (9.8)
Sex						
Male	60 (51.7)	36 (56.3)	24 (46.2)	552 (62.7)	272 (63.7)	280 (61.7)
Female	56 (48.3)	28 (43.8)	28 (53.9)	329 (37.3)	155 (36.3)	174 (38.3)
Blood pressure at entry, mean (SD), mm Hg						
Systolic	168.0 (15.8)	167.1 (15.3)	169.2 (16.5)	162.9 (14.2)	162.6 (14.2)	163.1 (14.3)
Diastolic	92.5 (10.9)	91.9 (9.8)	93.2 (12.2)	91.4 (9.4)	91.4 (9.2)	91.5 (9.6)
Baseline NIHSS score, median (IQR)	3.5 (2 to 5)	4 (2 to 6)	3 (2 to 5)	3 (2 to 5)	3 (2 to 5)	3 (2 to 5)
Medical history						
Hypertension	96 (82.8)	48 (75.0)	48 (92.3)	689 (78.2)	329 (77.1)	360 (79.3)
Diabetes	26 (22.4)	19 (29.7)	7 (13.5)	215 (24.4)	105 (24.6)	110 (24.2)
Dyslipidemia	3 (2.6)	1 (1.6)	2 (3.9)	27 (3.1)	12 (2.8)	15 (3.3)
Previous stroke	26 (22.4)	10 (15.6)	16 (30.8)	179 (20.3)	87 (20.4)	92 (20.3)
Previous TIA	2 (1.7)	2 (3.1)	0	6 (0.7)	1 (0.2)	5 (1.1)
Coronary heart disease	9 (7.8)	5 (7.8)	4 (7.7)	55 (6.2)	30 (7.0)	25 (5.5)
Current smoking	25 (21.7)	14 (21.9)	11 (21.6)	313 (35.5)	160 (37.5)	153 (33.7)
Previous medications use						
Antihypertensive therapy	71 (61.2)	34 (53.1)	37 (71.2)	458 (52.0)	206 (48.2)	252 (55.5)
Antiplatelet therapy	8 (6.9)	3 (4.7)	5 (9.6)	91 (10.3)	50 (11.7)	41 (9.0)
Lipid-lowering therapy	7 (6.0)	4 (6.3)	3 (5.8)	57 (6.5)	25 (5.9)	32 (7.1)
Time from onset to randomization, median (IQR), h	40.0 (30.7 to 45.6)	40.1 (29.5 to 45.9)	40.0 (31.9 to 45.1)	37.1 (29.8 to 45.0)	37.0 (30.0 to 45.0)	37.1 (29.5 to 45.2)
Time from onset to intervention, median (IQR), d	1.9 (1.6 to 8.5)	1.7 (1.2 to 1.9)	8.7 (8.3 to 8.8)	2.0 (1.5 to 8.4)	1.5 (1.2 to 1.9)	8.6 (8.2 to 8.9)
Time from onset to imaging, median (IQR), h	31.8 (22.0 to 54.1)	31.3 (20.5 to 48.8)	31.8 (23.0 to 66.8)	30.0 (20.0 to 53.0)	30.0 (19.0 to 52.0)	29.9 (21.0 to 54.0)
Time from randomization to imaging, median (IQR), h	−3.0 (−18.0 to 20.5)	−4.0 (−18.0 to 8.9)	−0.7 (−17.1 to 23.3)	−4.0 (−19.0 to 17.0)	−4.2 (−19.8 to 16.1)	−3.5 (−19.0 to 17.0)

Abbreviations: NIHSS, National Institutes of Health Stroke Scale; PAD, parent artery disease; SSI, single subcortical infarction; TIA, transient ischemic attack.

### Blood Pressure Reduction

**Figure 1.**
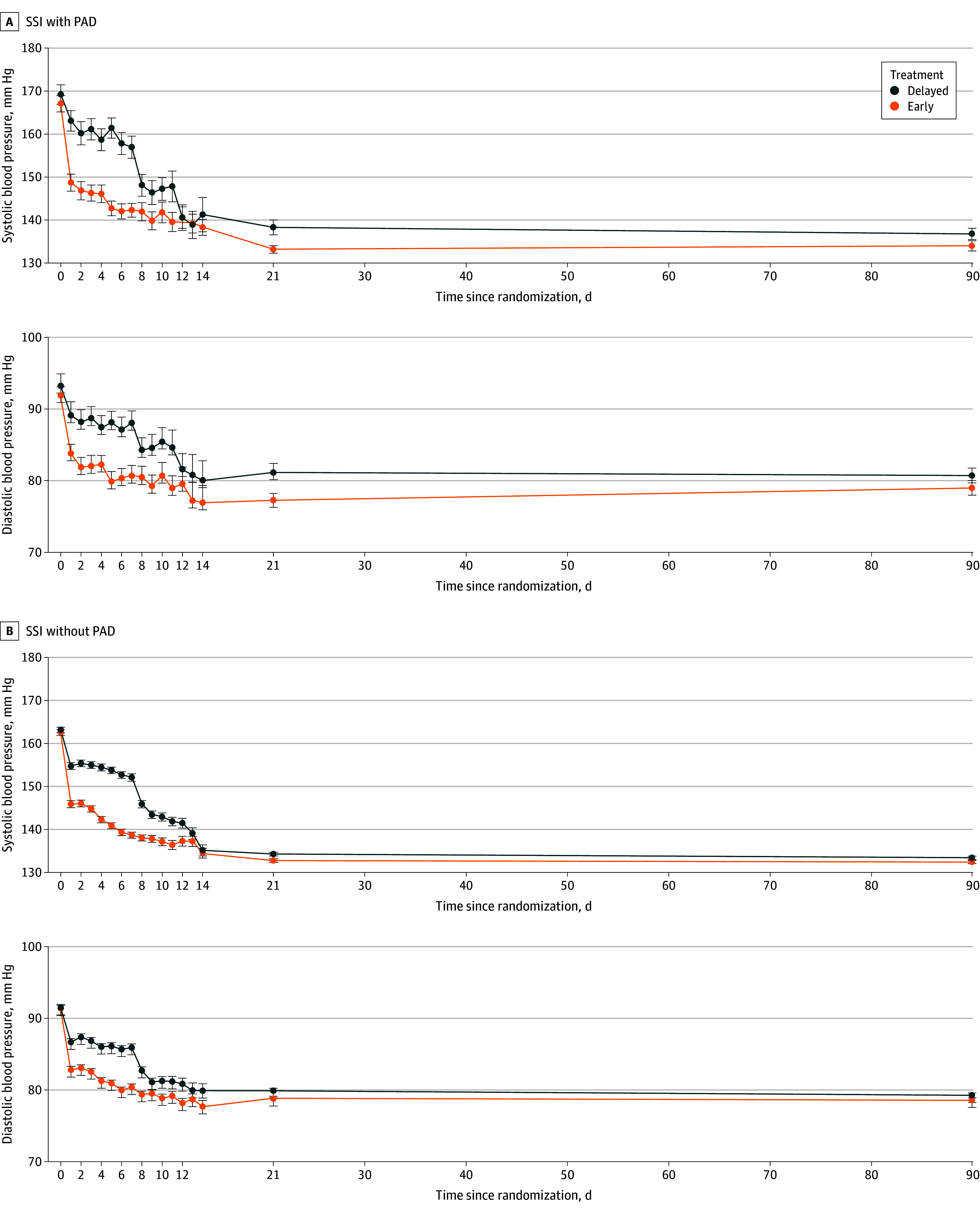
Changes in Mean Systolic and Diastolic Blood Pressure Since Randomization in Patients With Single Subcortical Infarction (SSI) With and Without Parent Artery Disease (PAD) Stenosis

BP reduction at different time points in early and delayed treatment groups in patients with SSI with and without PAD stenosis are presented in Table 2 and Figure 1. Within 24 hours after randomization, mean SBPs were decreased by 18.4 mm Hg (10.7%) and 16.7 mm Hg (9.9%) in the early treatment group, and 5.9 mm Hg (3.0%) and 8.5 mm Hg (4.9%) in the delayed treatment group for patients with SSI with PAD and SSI without PAD (*P* < .001 for group difference), respectively. The SBP differences between the 2 treatment groups were −14.7 (95% CI, −20.5 to −8.9) and −13.4 (95% CI, −15.6 to −11.2) mm Hg at day 7, and narrowed to −5.1 (95% CI, −8.8 to −1.5) and −1.5 (95% CI, −2.7 to −0.4) mm Hg at day 21, further narrowed down to −2.8 (95% CI, −6.3 to 0.7) and −1.0 (95% CI, −2.1 to 0.2) mm Hg at day 90 for patients with SSI with and without PAD stenosis, respectively. The antihypertensive medications used are summarized in eTable 3 in Supplement 1.

**Table 2.** Blood Pressure in Patients With SSI With and Without PAD Stenosis

	SSI with PAD				SSI without PAD			
	BP, mean (SD), mm Hg		BP difference (95% CI)	*P* value	BP, mean (SD), mm Hg		BP difference (95% CI)	*P* value
	Early treatment	Delayed treatment			Early treatment	Delayed treatment		
**BP at 24 h after randomization**								
Systolic	148.7 (15.8)	163.1 (17.1)	−14.4 (−20.5 to −8.3)	<.001	145.9 (16.6)	154.7 (16.3)	−8.8 (−11.0 to −6.6)	<.001
Diastolic	83.8 (10.3)	89.1 (13.7)	−5.3 (−9.7 to −0.9)	.02	82.8 (10.2)	86.7 (10.5)	−3.9 (−5.3 to −2.5)	<.001
**Absolute BP changed from baseline to 24 h after randomization**								
Systolic	−18.4 (14.8)	−5.9 (18.8)	−12.5 (−18.7 to −6.3)	<.001	−16.7 (17.0)	−8.5 (16.2)	−8.5 (−10.0 to −6.9)	<.001
Diastolic	−8.1 (9.2)	−4.1 (11.3)	−4.0 (−7.8 to −0.2)	.04	−8.6 (10.1)	−4.8 (10.3)	−4.8 (−5.8 to −3.9)	<.001
**Proportional BP changes from baseline to 24 h after randomization** ^a^								
Systolic	−10.7 (8.7)	−3.0 (10.4)	−7.7 (−11.3 to −4.2)	<.001	−9.9 (10.0)	−4.9 (9.7)	−4.9 (−5.8 to −3.9)	<.001
Diastolic	−8.5 (9.5)	−3.9 (11.9)	−4.6 (−8.6 to −0.7)	.02	−9.1 (10.7)	−4.8 (11.2)	−4.8 (−5.8 to −3.8)	<.001
**BP at day 7 after randomization**								
Systolic	142.3 (12.0)	157.0 (17.6)	−14.7 (−20.5 to −8.9)	<.001	138.7 (13.2)	152.1 (17.3)	−13.4 (−15.6 to −11.2)	<.001
Diastolic	80.7 (10.9)	88.0 (11.6)	−7.4 (−11.8 to −3.0)	<.001	80.4 (9.1)	85.9 (10.9)	−5.5 (−7.0 to −4.1)	<.001
**BP at day 21 after randomization**								
Systolic	133.2 (6.9)	138.3 (12.5)	−5.1 (−8.8 to −1.5)	.01	132.8 (8.8)	134.3 (8.9)	−1.5 (−2.7 to −0.4)	.01
Diastolic	77.3 (7.4)	81.1 (9.2)	−3.9 (−6.9 to −0.8)	.01	78.8 (7.4)	79.9 (7.7)	−1.1 (−2.1 to −0.1)	.03
**BP at day 90 after randomization** ^b^								
Systolic	134.0 (9.3)	136.8 (9.4)	−2.8 (−6.3 to 0.7)	.12	132.4 (8.3)	133.4 (8.9)	−1.0 (−2.1 to 0.2)	.10
Diastolic	79.0 (8.2)	80.7 (7.7)	−1.7 (−4.7 to 1.3)	.26	78.6 (7.2)	79.2 (7.2)	−0.7 (−1.7 to 0.3)	.16

Abbreviations: BP, blood pressure; PAD, parent artery disease; SSI, single subcortical infarction.

^a^ These proportional changes are measured as percentages.

^b^ Missing data (n = 24).

### Clinical Outcomes in All Patients With SSI 

The primary outcome of functional dependency or death at 90 days occurred in 79 patients (7.9%), which was not significantly different between the early and delayed treatment groups (8.8% vs 7.1%; OR, 1.25 [95% CI, 0.79-1.99]; *P* = .34) (eTable 4 in Supplement 3). Similar results were observed in all secondary outcomes. Multiple imputation of missing primary outcome generated consistent results (OR, 1.28 [95% CI, 0.81-2.03]; *P* = .29). Similar results among all clinical outcomes were also observed in patients with SSI with infarction diameter less than or equal to 20 mm and greater than 20 mm (eTable 5 in Supplement 3).

### Clinical Outcomes in Patients With SSI With and Without PAD Stenosis

**Figure 2.**
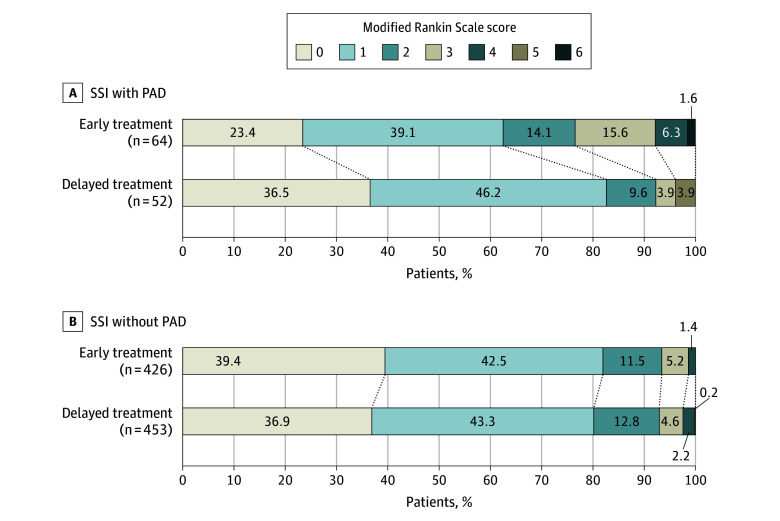
Distribution of Modified Rankin Scale Scores at 90 Days in Patients With Single Subcortical Infarction (SSI) With and Without Parent Artery Disease (PAD) Stenosis

Among patients with SSI with PAD, early antihypertensive treatment was associated with increased risk of the primary outcome compared with delayed treatment (23.4% vs 7.7%; OR, 3.67 [95% CI, 1.14-11.86]; *P* = .03) (Table 3). In addition, there was a significant difference favoring the delayed treatment group over the early treatment group in the overall distribution of mRS scores in patients with SSI with PAD (OR, 2.30 [95% CI, 1.16-4.58]; *P* = .02) (Table 3 and Figure 2). Among patients with SSI without PAD, the risks of the primary outcome (6.6% vs 7.1%; OR, 0.93 [95% CI, 0.55-1.57]; *P* = .77) (Table 3) and the distribution of mRS scores (OR, 0.89 [95% CI, 0.70-1.14]; *P* = .37) (Table 3 and Figure 2) were comparable between early and delayed antihypertensive treatment groups. For the treatment × presence of PAD stenosis interaction analyses, a significant interaction was detected for the primary outcome (*P* for interaction = .04), and for the distribution of mRS scores (*P* for interaction = .01). There was no significant difference in other secondary outcomes including recurrent stroke, major vascular events, and END between the early and delayed treatment groups in patients with SSI with PAD and SSI without PAD.

**Table 3.** Early Vs Delayed Antihypertensive Treatment on Clinical Outcomes in Patients With SSI With and Without PAD Stenosis

Outcomes	With PAD				Without PAD				*P* value for interaction
	Patients with SSI, No./total No. (%)		OR (95% CI)	*P* Value	Patients with SSI, No./total No. (%)		OR (95% CI)	*P* value	
	Early treatment	Delayed treatment			Early treatment	Delayed treatment			
Primary outcome									
Functional dependency or death at 90 d (mRS score ≥3)	15/64 (23.4)	4/52 (7.7)	3.67 (1.14-11.86)	.03	28/426 (6.6)	32/453 (7.1)	0.93 (0.55-1.57)	.77	.04
Secondary outcome									
Ordinal mRS scores at 90 d	NA	NA	2.30 (1.16-4.58)^a^	.02	NA	NA	0.89 (0.70-1.14)^a^	.37	.01
0	15/64 (23.4)	19/52 (36.5)	NA	NA	168/426 (39.4)	167/453 (36.9)	NA	NA	NA
1	25/64 (39.1)	24/52 (46.2)	NA	NA	181/426 (42.5)	196/453 (43.3)	NA	NA	NA
2	9/64 (14.1)	5/52 (9.6)	NA	NA	49/426 (11.5)	58/453 (12.8)	NA	NA	NA
3	10/64 (15.6)	2/52 (3.9)	NA	NA	22/426 (5.2)	21/453 (4.6)	NA	NA	NA
4	4/64 (6.3)	0	NA	NA	6/426 (1.4)	10/453 (2.2)	NA	NA	NA
5	0	2/52 (3.9)	NA	NA	0	1/453 (0.2)	NA	NA	NA
6	1/64 (1.6)	0	NA	NA	0	0	NA	NA	NA
Recurrent stroke	6/64 (9.4)	3/52 (5.8)	1.69 (0.40-7.11)	.47	25/427 (5.9)	23/454 (5.1)	1.17 (0.65-2.09)	.61	.64
Major vascular events	6/64 (9.4)	3/52 (5.8)	1.69 (0.40-7.11)	.47	25/427 (5.9)	26/454 (5.7)	1.02 (0.58-1.80)	.94	.52
END	5/64 (7.8)	4/52 (7.7)	1.02 (0.26-4.00)	.98	14/427 (3.3)	16/453 (3.5)	0.93 (0.45-1.92)	.84	.91

Abbreviations: END, early neurological deterioration; mRS, modified Rankin Scale; NA, not applicable; OR, odds ratio; PAD, parent artery disease; SSI, single subcortical infarction.

^a^ Odds of a 1-unit higher mRS score.

### Sensitivity Analysis

After adjusting for potential confounders, early antihypertensive treatment remained associated with increased risk of the primary outcome compared with delayed treatment among patients with SSI with PAD (adjusted OR, 4.78 [95% CI, 1.19-19.16]; *P* = .03) (eTable 6 in Supplement 3); this finding was not observed among patients with SSI without PAD (adjusted OR, 1.09 [95% CI, 0.61-1.92]; *P* = .78). The heterogeneity of treatment association with presence of PAD stenosis with the primary outcome was attenuated (*P *for interaction = .08). The results of sensitivity analysis for treatment association with all secondary outcomes were consistent. Similar results among all clinical outcomes were also observed in patients with SSI with and without PAD stenosis evaluated by MRA or computed tomography angiography (eTable 7 in Supplement 3).

## Discussion

In this subgroup analysis of the CATIS-2 trial, we did not observe an association of early vs delayed antihypertensive treatment with clinical outcomes within 3 months, across patients with acute SSI. However, the outcomes were different in those with and without coexisting PAD stenosis. Early antihypertensive treatment was associated with an increased risk of functional dependency or death at 90 days compared with delayed treatment particularly in patients with SSI with PAD stenosis, while there was no difference between early and delayed treatment in those without PAD stenosis. Our study highlights that while CATIS-2 shows that acute BP lowering is not beneficial in any AIS, it may be particularly detrimental in SSI with PAD stenosis.

The prevalence of coexisting PAD among patients with SSI in previous studies varied from 10% to 30%,^16,22,23,24,27^ while our study observed a prevalence of 11.8%. Different inclusion criteria and definitions regarding PAD may contribute to these inconsistent findings. Previous studies have shown different baseline characteristics and outcomes between patients with SSI with and without PAD.^16,28^ Our current study showed that patients with SSI with PAD stenosis were on average older, more likely to be female, less likely to be current smokers, who had higher baseline SBP, in line with previous findings.^22,27,29^ These results suggest possibly different stroke mechanisms between patients with SSI with and without PAD.

Recent guidelines from the European Stroke Organization highlight the ongoing uncertainty regarding BP lowering therapy in such patients.^30^ Several clinical trials have evaluated the effects of immediate antihypertensive treatment vs no intervention or specific antihypertensive agents vs placebo on clinical outcomes in patients with AIS,^31,32,33,34,35^ with secondary analyses across patients with different stroke subtypes.^7,36,37,38^ A meta-analysis of these studies found that immediate antihypertensive treatment, compared to avoiding intervention, did not show more benefits in patients with acute lacunar stroke compared to AIS patients in general.^30^ However, the optimal time for initiating antihypertensive treatment among patients with SSI was not considered in these studies. Our findings further show that delaying antihypertensive treatment to 8 days after stroke onset did not increase the risk of clinical outcomes in patients with SSI. In fact, early BP reduction may lead to worse functional outcomes in those with PAD stenosis, suggesting that delayed treatment for all patients with SSI might be the clearest strategy going forward.

Additionally, the different responses to early antihypertensive treatment between patients with SSI with and without PAD stenosis may be due to different potential mechanisms. With more pronounced cerebral autoregulation impairment, rapid BP fluctuations during acute phase might exacerbate cerebral ischemia by passively reducing cerebral perfusion,^12,39,40,41^ leading to poorer neurological outcomes for patients with SSI with PAD stenosis. Furthermore, the outcome of END and recurrent stroke was similar between the 2 treatments in patients with SSI. More work is needed to better understand the mechanisms underlying these findings, probably by involving cerebral perfusion and hemodynamics in the investigations.

### Limitations

This study had limitations. First, potential selection bias should be considered because CATIS-2 patients who did not undergo MRI scans during hospitalization were excluded from current analyses despite the scans being conducted regardless of treatment allocation. Second, the current study does not provide any information on the clinical features of the SSI so as to aid use of early clinical diagnosis in acute BP management. Additionally, our study is unable to provide insights into whether the degree of stenosis modifies outcomes due to the small sample size. Further research on these aspects would be valuable. Third, a relatively small sample size of the SSI with PAD group may result in a wide confidence interval and low precision. The findings from this subgroup analysis should be interpreted with caution in the overall population. Fourth, some potential confounders (eg, age and baseline NIHSS scores) need to be considered. After adjusting for these factors, the treatment association was attenuated, which may be related to the limited sample size or suggest that part of the differences in outcomes might be due to the different demographics. Fifth, without vessel wall imaging, we cannot detect nonstenotic PAD (eg, diffuse atheromatous wall involvement without substantial focal narrowing). Further studies with magnetic resonance vessel wall imaging will more accurately detect the relevance of PAD to a SSI lesion, which can further verify the associations of early and delayed antihypertensive therapies in patients with SSI with different etiologies.

## Conclusions

This secondary analysis of the CATIS-2 trial indicates no association of early vs delayed antihypertensive treatment with clinical outcomes within 3 months, across patients with acute SSI. However, there was a significant treatment-by-presence of PAD stenosis interaction. Early antihypertensive treatment was associated with an increased risk of death or functional dependency at 90 days compared with delayed treatment among patients with SSI with PAD stenosis, which was not observed in patients with SSI without PAD stenosis. Studies are warranted to explore the underlying mechanisms of our findings and to test for more individualized BP management strategy in patients with SSI.

## Notes

Supplement 1.
Trial Protocol

Supplement 2.
Statistical Analysis Plan

Supplement 3. **eTable 1.** Baseline Characteristics Between Patients With and Without Brain MRI Scans During Hospitalization **eTable 2.** Baseline Characteristics of the Included Patients According to 2 Treatment Arms **eTable 3.** Use of Antihypertensive Medications During Follow-Up in SSI Patients With and Without PAD Stenosis **eTable 4.** Clinical Outcomes Between Early and Delayed Antihypertensive Treatment Among Patients With SSI **eTable 5.** Clinical Outcomes Between Early and Delayed Antihypertensive Treatment Among Patients With SSI Stratified by Infarction Diameter **eTable 6.** The Sensitivity Analysis of 2 Treatment Arms on Clinical Outcomes in SSI Patients With and Without PAD Stenosis **eTable 7.** Early Versus Delayed Antihypertensive Treatment on Clinical Outcomes in Patients With PAD Stenosis Evaluated by MRA or CTA **eFigure 1.** Examples of Single Subcortical Infarction (SSI) With or Without Parent Artery Disease (PAD) Stenosis **eFigure 2.** Flowchart of the Current Study

Supplement 4.
Data Sharing Statement
