# Supplementary material for: Early vs Delayed Antihypertensive Treatment in Acute Single Subcortical Infarction: A Secondary Analysis of the CATIS-2 Randomized Clinical Trial
Source: JAMA Netw Open. 2024 Aug 30;7(8):e2430820. doi: 10.1001/jamanetworkopen.2024.30820 (PMC11365005; doi:10.1001/jamanetworkopen.2024.30820)
Supplement: Supplement 1. — Trial Protocol [file jamanetwopen-e2430820-s001.pdf]

China Antihypertensive Trial in Acute Ischemic Stroke II (CATIS-2)

Study Protocol

Version 3.0

CATIS-2 Study Steering Committee

October 2018

Beijing • New Orleans

CATIS-2

China Antihypertensive Trial in Acute Ischemic Stroke II

Leading Institutes:

Beijing Tiantan Hospital, Capital Medical University, Beijing, China

Tulane University School of Public Health and Tropical Medicine, New Orleans, LA, USA

Suzhou Medical College of Soochow University School of Public Health, Suzhou, China

Contact Information:

Department of Neurology

Beijing Tiantan Hospital, Capital Medical University

119 South 4th Ring West Road, Fengtai District

Beijing 100070, China.

Tel: (86 10) 5997-5097

Fax: (86 10) 5997-3383

Email: catis_2@163.com

Executive Summary

Stroke is the leading cause of death in China and the second leading cause of death in the world. We propose to conduct a multicenter randomized trial to test the primary hypothesis of whether early antihypertensive treatment starting between the first 24-48 hours after the onset of an acute ischemic stroke will reduce the risk of composite case-fatality and major disability (modified Rankin Scale score ≥3) at three months compared to delayed antihypertensive treatment (starting on day 8 after stroke onset). In the proposed China Antihypertensive Trial in Acute Ischemic Stroke II (CATIS-2) study, we will recruit 4,776 patients from 100 hospitals within the China Stroke Clinical Research Network. Eligibility criteria for the trial participants include age ≥40 years, acute ischemic stroke confirmed by computed tomography or magnetic resonance imaging, symptom onset between 24-48 hours, and average systolic blood pressure (BP) between 140 to <200 mmHg at randomization. Patients with extracranial or intracranial artery stenosis (≥70%) in both sides or the affected side, NIH Stroke Scale score of ≥21, Glasgow Coma Scale score <8, preceding moderate or severe dependency (modified Rankin scale score 3-5), revascularization, intravenous thrombolytic therapy (i.e., intravenous recombinant tissue plasminogen activator [rtPA]) or mechanical thrombectomy will be excluded. All eligible patients will discontinue their home antihypertensive medications. Patients admitted within 24 hours of symptom onset will require a reevaluation prior to randomization at 24 hours after stroke onset. After randomization, patients in the early treatment group will immediately receive antihypertensive treatment aimed at lowering average systolic BP by 10-20% within the first 24 hours and achieving an average BP <140/90 mmHg within seven days. Patients in the delayed treatment group will discontinue antihypertensive medications for the first seven days. After seven days, both groups will receive antihypertensive treatment with a BP goal of <140/90 mmHg. The primary study endpoint is a composite outcome of death and major disability (modified Rankin Scale score ≥3) at three months. The major secondary endpoint will be the first recurrent stroke (hemorrhagic or ischemic) over three-month follow-up after randomization. Other secondary endpoints include ordered 7-level categorical score of the modified Rankin Scale, all-cause mortality, and major vascular events at three months. The proposed study provides 85% statistical power to detect a 15% reduction in the composite outcome of death and major disability over three months at a significance level of 0.05 for a two-sided test. Based on experience from our previous trials, we assumed a 25% event rate of the primary study endpoint and potential loss to follow-up of 5% over three months. The CATIS-2 trial will provide important information for the development of clinical guidelines in the early management of BP among patients with acute ischemic stroke for reducing mortality and major disability.

A. Specific Aims

Stroke is the leading cause of death in China and the second leading cause of death in the world.[1-3] In addition, stroke is the leading cause of long-term disability worldwide and a major financial burden to society. It is estimated that there are probably more than 3 million new strokes every year in China.[4] In 2013, more than 1.9 million Chinese adults died from stroke, which represented an increase of 47.7% from 1.3 million in 1990.[1]

Elevated blood pressure (BP) is common in the acute phase of ischemic stroke, occurring in ≥75% of all patients.[5,6] The early BP increase following ischemic stroke often reflects uncontrolled chronic hypertension. In addition, the potential causes of this transient rise include disturbed cerebral autoregulation, damage or compression of brain regions that regulate BP, neuroendocrine disturbance, and non-specific mechanisms such as headache, urine retention, and psychological stress.[7,8] Several clinical trials have tested the effects of immediate BP lowering on adverse clinical outcomes in patients with acute ischemic stroke and showed a neutral effect on death or dependency.[9-12] The China Antihypertensive Trial in Acute Ischemic Stroke (CATIS) suggested a beneficial effect of BP lowering on death or major disability (odds ratio [OR] 0.73, 95% confidence interval [CI] 0.55-0.96, p=0.03) and recurrent stroke (OR 0.25, 95% CI 0.08-0.74, p=0.01) among patients with acute ischemic stroke who received antihypertensive treatment between 24-48 hours in a subgroup analysis.[11,13] There are important knowledge gaps in BP management in acute ischemic stroke, such as when is the optimal time for initiation of antihypertensive treatment.

We propose to conduct a multicenter randomized controlled trial to test the primary hypothesis of whether early antihypertensive treatment starting between the first 24-48 hours after the onset of an acute ischemic stroke will reduce the risk of composite case-fatality and major disability (modified Rankin scale score ≥3) at three months compared to delayed antihypertensive treatment (starting on day 8 after stroke onset). Patients with acute ischemic stroke will be randomly assigned within the first 24-48 hours after stroke onset to the early and delayed treatment groups. Patients in the early treatment group will immediately receive antihypertensive treatment aimed at lowering average systolic BP by 10-20% (with a mean reduction of 15%) within the first 24 hours, achieving an average systolic/diastolic BP <140/90 mmHg within seven days, and maintaining this level afterward. Patients in the delayed treatment group will discontinue antihypertensive medications for the first seven days. After then, both groups will receive antihypertensive treatment with an average systolic/diastolic BP goal of <140/90 mmHg. The primary study endpoint will be a composite outcome of death and major disability (modified Rankin Scale score ≥3) at three months after randomization. The major secondary endpoint will be the first recurrent stroke (hemorrhagic or ischemic) within three months. Other secondary endpoints include ordered 7-level categorical score of the modified Rankin Scale, all-cause mortality, and major vascular events at three months.

In the proposed China Antihypertensive Trial in Acute Ischemic Stroke II (CATIS-2), we will recruit 4,776 patients (2,388 for each group) from 100 hospitals within the China Stroke Clinical Research Network. Eligibility criteria for the trial participants include age ≥40 years, acute ischemic stroke confirmed by computed tomography (CT) or magnetic resonance imaging (MRI), symptom onset between 24-48 hours, and an average systolic BP between 140 to <200 mmHg. The proposed study provides 85% statistical power to detect a 15% reduction in the primary study endpoint at three months at a significance level of 0.05 for a two-sided test. Based on experience from our previous CATIS trial, we assumed an event rate of 25% for the primary study outcome and potential loss to follow-up of 5% over three months.[11] To achieve the CATIS-2 study objectives, we plan to:

1. Recruit and randomize 4,776 eligible patients (2,388 for each group) to the early antihypertensive

treatment group or the delayed antihypertensive treatment group according to the study eligibility criteria;

2. Achieve and maintain target treatment goals for BP in the randomization groups;

3. Follow a study-wide strategy to encourage a standard of care based on clinical guidelines for the treatment of acute ischemic stroke other than BP for all participants;

4. Obtain follow-up data on clinical endpoints, neurological, and functional status for each participant according to the study protocol; and

5. Estimate the effect of early antihypertensive treatment vs. delayed treatment on the primary and secondary endpoints according to intention-to-treat analysis and conduct predefined subgroup analyses.

The CATIS-2 study will provide important information for the development of clinical guidelines in the early management of BP among patients with acute ischemic stroke for reducing mortality and major disability.

B. Significance and Scientific Premise

B1. Stroke as a major global public health challenge

Stroke is a major global public health challenge due to its high morbidity and mortality, consequent serious long-term disability, and financial burden to society.[1-3,14] Stroke is the second most common cause of death worldwide.[2,3,14] Based on estimates from the Global Burden of Disease Study 2013, there were 25.7 million prevalent strokes (18.3 million ischemic strokes and 7.4 million hemorrhagic strokes), 10.3 million incident strokes (6.9 million ischemic strokes and 3.4 million hemorrhagic strokes), and 6.4 million stroke deaths (3.3 million ischemic stroke deaths and 3.2 million hemorrhagic stroke deaths) worldwide in 2013, which reflected an 83.6% increase in prevalent cases, 65.6% increase in incident cases, and 40.2% increase in stroke deaths from 1990.[2,3,14] In 2013, stroke accounted for 12% of all deaths globally.[2] Approximately 4.85 million stroke deaths occurred in low- and middle-income countries and 1.6 million deaths in high-income countries.[3] Stroke is the leading cause of death and the leading cause of long-term disability in China.[1,15] In 2013, more than 1.9 million Chinese adults died from stroke, which represented an increase of 47.7% from 1.3 million in 1990.[3]

B2. Antihypertensive treatment in acute ischemic stroke

Elevated blood pressure (BP) is common in the acute phase of ischemic stroke, occurring in about 75% of all patients.[7,8] The early BP increase following ischemic stroke often reflects uncontrolled or undiagnosed chronic hypertension. In addition, the potential causes of this transient rise include disturbed cerebral autoregulation, damage or compression of brain regions that regulate BP, neuroendocrine disturbance, and non-specific mechanisms such as headache, urine retention, and psychological stress.[7,8] Several large clinical trials have tested the effects of BP lowering on adverse clinical outcomes in patients with acute ischemic stroke and showed a neutral effect on death or dependency.[9-11] The Scandinavian Candesartan Acute Stroke Trial (SCAST) randomly allocated 2,029 patients with acute stroke (ischemic or hemorrhagic) and systolic BP of ≥140 mmHg within 30 hours of symptom onset to candesartan or placebo for 7 days.[9] During a 6-month follow-up, the composite vascular endpoint did not differ between treatment groups (hazard ratio 1.09, 95% CI 0.84–1.41; p=0.52). A higher risk of poor functional outcome in the candesartan group was suggested (common OR 1.17, 95% CI 1.00-1.38; p=0.048 [not significant at p≤0.025 level]). The Efficacy of Nitric Oxide in Stroke (ENOS) trial randomly assigned 4,011 patients with an acute ischemic or hemorrhagic stroke and systolic BP of 140–220 mmHg to 7

days of transdermal glyceryl trinitrate or to no glyceryl trinitrate.[10] A subset of 2,097 patients who were taking antihypertensive drugs before their stroke were also randomly assigned to continue or stop taking antihypertensive drugs. Functional outcome at day 90 did not differ in either treatment comparison: OR for glyceryl trinitrate was 1.01 (95% CI 0.91–1.13; p=0.83), and OR for continued antihypertensive drug use was 1.05 (0.90–1.22; p=0.55). CATIS randomly assigned 4,071 patients with acute ischemic stroke within 48 hours of onset and elevated systolic BP to receive antihypertensive treatment or to discontinue all antihypertensive medications.[11] The primary outcome of death and major disability did not differ between treatment groups (OR=1.00, 95% CI, 0.88–1.14; P=0.98) at 14 days or hospital discharge. These large trials, as well as several small trials, were included in a meta-analysis which showed that BP lowering in early ischemic stroke did not affect the risk of death or dependency at 3 months (relative risk [RR], 1.04; 95% CI 0.96-1.13; P=0.35).[12]

Antihypertensive treatment was initiated within 24 hours among all of these trials.[9-11] The CATIS trial suggested a beneficial effect of BP lowering on death or major disability (OR 0.73, 95% CI 0.55-0.96, p=0.03), recurrent stroke (OR 0.25, 95% CI 0.08-0.74, p=0.01), and vascular events (OR 0.41, 95% CI 0.18-0.95, p=0.04) among patients with acute ischemic stroke who received treatment between 24-48 hours in a subgroup analysis.[11,13] This subgroup analysis generated hypotheses that BP lowering initiated between 24-48 hours after stroke onset might reduce risk of death or major disability, recurrent stroke, and vascular events among patients with acute ischemic stroke.

B3. Knowledge gaps in early BP management of minor ischemic stroke

The American Heart Association (AHA)/American Stroke Association (ASA) guidelines acknowledge that "the optimal time after the onset of acute ischemic stroke to restart or start long-term antihypertensive therapy has not been established".[16] It was suggested that BP reduction in the first 24 hours of stroke onset was associated with poor outcome at 3 months in observational studies.[17] The CATIS trial also found that BP lowering within 12 hours after stroke onset increased deaths (OR 3.40, 95% CI 1.09-10.6, P=0.03) and vascular events (OR 5.76, 95% CI 1.26-26.3, P=0.02) among acute ischemic stroke patients without a history of hypertension. Experts have recommended not starting antihypertensive drugs within the first 24 hours[16] or within the first seven days of acute ischemic stroke.[18] However, no clinical trial has tested the optimal time for early BP lowering in acute ischemic stroke.

B4. Rationale for the proposed CATIS-2

Whether and when to lower BP among patients with acute ischemic stroke are the major unresolved issues in management of acute stroke. Theoretically, moderate arterial hypertension during acute ischemic stroke might be advantageous by improving cerebral perfusion of the ischemic tissue, or it might be detrimental by exacerbating edema and hemorrhagic transformation of the ischemic tissue.[16,19,20] An early hypertensive response to physical and psychological stresses from brain ischemia is an important contributing factor for elevated BP in acute ischemic stroke. This initial hypertensive response is self-limiting, most marked in the first few hours following the onset of cerebral ischemia, and resolving over several days.[7,8,20] Therefore, an optimal strategy for management of BP might be to avoid BP-lowering agents during the first 24 hours after stroke onset, when collateral circulation compromise is still a substantial concern in most patients, and then to implement BP lowering in the 24-48 hours after onset to help avert secondary injury and ensure that the patient will be transitioned to long-term antihypertensive therapy for secondary prevention.[16,20] The CATIS time-to-randomization subgroup analysis suggests better 3-month outcomes may result if BP lowering is started beyond

24 hours.[11] However, subgroup analyses can only generate hypotheses which should be tested in sufficiently powered randomized controlled trials. The proposed CATIS-2 trial will provide evidence to guide clinical care of acute ischemic stroke patients with elevated BP for reducing mortality and major disability.

B5. Clinical and Public Health Impacts

BP management in acute ischemic stroke is a critically important and controversial issue. Clinical trials have indicated that immediate BP lowering in patients with acute ischemic stroke did not increase or reduce mortality and major disability.[9-11] The CATIS trial subgroup analysis suggested that BP lowering after 24 hours among patients with acute ischemic stroke and elevated BP might be beneficial.[11] The proposed CATIS-2 trial will test the efficacy and safety of early antihypertensive treatment starting between the first 24-48 hours compared to delayed antihypertensive treatment for the first seven days after stroke onset on mortality and major disability in patients with an acute ischemic stroke. The CATIS-2 Trial will answer the important clinical questions of whether and when to initiate antihypertensive treatment and provide important information for developing clinical guidelines for the early management of BP among patients with acute ischemic stroke.

B6. Innovation

The proposed CATIS-2 trial is novel in several aspects:

- The CATIS-2 trial is the first randomized clinical trial to compare the effect of early antihypertensive treatment within the first 24-48 hours after stroke onset and delayed treatment for 7 days after stroke onset on deaths and major disability among patients with acute ischemic stroke.
- The CATIS-2 trial is the largest randomized clinical trial in patients with acute stroke to test the efficacy and safety of early BP lowering on mortality and major disability. The CATIS-2 trial has sufficient statistical power to detect a 15% reduction in recurrent stroke over three months of follow-up.
- The CATIS-2 trial represents a true collaboration among US and Chinese clinical investigators.

C. Study Design, Timeline, and Management

C1. Overview of trial design

The proposed CATIS-2 trial is a multicenter, randomized, open-label, blinded-endpoints trial that will be conducted in 100 hospitals within the China Stroke Clinical Research Network. The CATIS-2 trial is designed to test whether early antihypertensive treatment starting between the first 24-48 hours after the onset of an acute ischemic stroke, compared to delayed antihypertensive treatment for the first seven days, will reduce the risk of mortality and major disability events, recurrent stroke, and cardiovascular disease (CVD) over three months. The primary study endpoint is the composite outcome of death and major disability within three months after randomization. The secondary endpoints are the first recurrent stroke (hemorrhagic or ischemic), modified Rankin score, all-cause mortality, and major vascular events over three months. The CATIS-2 trial will recruit 4,776 eligible patients (2,388 for each group) aged ≥40 years with acute ischemic stroke confirmed by CT or MRI onset between 24-48 hours and with systolic BP between 140-200 mmHg from hospitals within the China Stroke Clinical Research Network and randomly assign them to two parallel arms:

- Early antihypertensive treatment group: Initiate antihypertensive treatment within 24-48 hours of stroke onset to achieve the target systolic BP lowering by 10% to 20% within the first 24 hours after

randomization and to achieve systolic BP below 140 mmHg and diastolic BP below 90 mmHg within 7 days and to maintain this BP level afterward.

- Delayed antihypertensive treatment group: Discontinue all home antihypertensive medications within the first seven days after stroke onset. Initiate antihypertensive treatment on day eight after stroke onset and achieve systolic BP <140 mmHg and diastolic BP <90 mmHg within two weeks and maintain this BP level afterward.

Besides receiving or not receiving antihypertensive treatment during the first seven days of hospitalization, both groups will receive standard care according to AHA/ASA guidelines,[16] which is consistent with the Chinese National Guidelines for Prevention and Treatment of Cerebrovascular Diseases.[21]

C2. Timeline

The timeline and tasks of the CATIS trial are presented in **Table 1**. In the first six months, we will finalize the study protocol and manual of procedures (MOP). The comments from the Data and Safety Monitoring Board (DSMB) will be incorporated into the final study protocol and treatment plan. The study protocol, forms, and MOP will be translated into Chinese and pilot-tested. We will seek approval from IRBs at Tulane University in the US, Beijing Tiantan Hospital of Capital Medical University, Soochow University and participating hospitals in China. Participant recruitment will last for two years. The intervention and data collection, quality control, and entry will continue into year 3. We will conduct data analysis and disseminate trial results in year 4.

Table 1. Timeline

Tasks	Year 1 (01/17–12/17)		Year 2 (01/18–12/18)		Year 3 (01/19–12/19)		Year 4 (01/20–12/20)	
Developing protocol and MOP	X							
Training	X							
Recruitment		X	X	X	X			
Intervention		X	X	X	X	X		
Data collection		X	X	X	X	X		
Data entry and QC		X	X	X	X	X		
Analysis and dissemination							X	X

C3. Study Management and Communication

C3.1. Trial organization

The proposed study will be overseen by a study steering committee, which consists of investigators from the Study and Data Coordinating Center (SDCC) and participating hospitals. A DSMB will provide guidance regarding patient safety issues. The SDCC and 100 participating hospitals will conduct the trial (**Figure 1**).

The Steering Committee will provide overall leadership on the design, conduct, analysis, and reporting of the study. The committee will be co-chaired by co-PIs, and consist of investigators from all participating hospitals. The Steering Committee will meet twice per year to discuss and make decisions on important scientific and study management issues. An Executive Committee will oversee the trial operation on behalf of the entire steering committee.

Figure 1. Trial organization

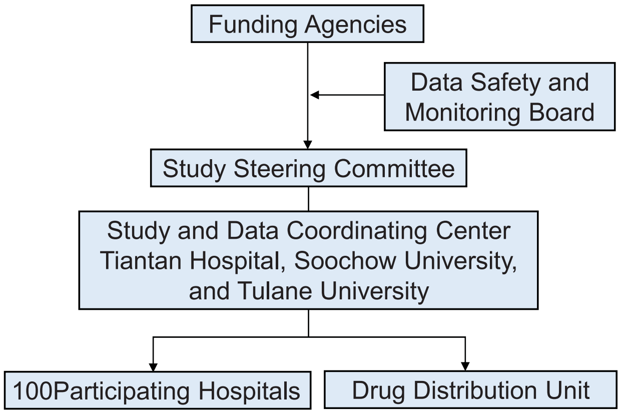

The SDCC will be co-directed by co-PIs at Beijing Tiantan Hospital and Soochow University in China and at Tulane University in the US. The SDCC will be staffed by personnel from the three institutes. The SDCC will work closely with the Steering Committee to provide scientific and administrative support for the trial. Specifically, the SDCC will coordinate activities related to finalizing the study protocol, study forms, and MOP; conduct centralized randomization; establish and maintain a secured computer database for data entry and management; provide training (retraining) in data collection, entry, and transmission; monitor patient recruitment, adherence, and retention; conduct ongoing data quality control; oversee the participating hospitals, drug distribution unit, and the central study laboratory; prepare and obtain IRB approval for the study protocol and related materials; arrange the Steering Committee meetings and DSMB meetings; facilitate communication among all organizational study components; conduct statistical analyses of the study data; and prepare scientific publications and data dissemination. We have worked efficiently in the previous multicenter clinical trials and epidemiological studies using this organizational model.

A Drug Distribution Unit at Beijing Tiantan Hospital will receive, package, and distribute all pharmaceuticals required for the proposed trial to the participating hospitals on a timely basis.

The primary responsibilities of participating hospitals are to obtain approval of the study protocol and informed consent documents from their own IRB or Ethics Committee, to recruit study participants who meet all eligibility criteria, to deliver the study treatment per randomization assignment, to follow-up study participants and collect study data according to the study protocol and MOP, to provide timely data on recruitment and measurements to the SDCC, and to capture and report major adverse drug effects in a timely fashion to maximize patient safety.

C3.2. Data and Safety Monitoring Board (DSMB)

The DSMB will be responsible for monitoring all aspects of the study, including those that require access to blinded data. The DSMB will consist of seven experts who are not otherwise affiliated with the study. The DSMB will meet at least once per year in-person or by Webinar conference calls. The proposed co-PIs will attend these meetings (but will not have a vote) and will be responsible for preparing and presenting up-to-date statistical reports on the progress of the study. The DSMB may recommend discontinuation of the trial for safety reasons or if there is a very low probability of successfully addressing the study hypotheses within the time frame. Funding agencies will make the final decision on whether or not to accept the DSMB's recommendation.

C3.3. Communication among study components

To facilitate communication among all organizational components within the proposed study, regularly scheduled meetings will take place. The Steering Committee will meet twice per year to discuss and make decisions on important scientific and management issues. The Steering Committee members will participate in monthly Webinar conference calls to discuss and make decisions on issues related to study operation. In addition, the Executive Committee will meet at weekly Webinar conference calls to deal with operational issues. The

conference calls usually take place in the morning in China and evening in the US to accommodate the time difference. Important information, such as recruitment yields, data completion and quality, and adherence to the protocol, will be sent to committee members and core staff by e-mail monthly. In addition, investigators will use e-mail communication frequently. The SDCC investigators and key staff will conduct regular site visits to each participating hospital for quality assurance.

D. Study Participant

The proposed trial will test the efficacy of early BP lowering on death and major disability and other clinical outcomes in patients with acute ischemic stroke. Patients with TIA will not be included because the proposed early or delayed antihypertensive treatment programs will last for seven days during hospitalization. Although stroke patients usually stay in the hospital for more than seven days in China, most TIA patients are discharged after an overnight hospital stay or emergency room observation.

D1. Eligibility criteria

The eligibility criteria for the CATIS-2 trial are listed in **Table 2**. The CATIS-2 trial will recruit patients with acute ischemic stroke onset between 24-48 hours confirmed by CT or MRI. Systolic BP >200 and diastolic BP >120 mmHg have been recommended for antihypertensive treatment in acute ischemic stroke by published clinical guidelines.[16,21] In the CATIS-2 trial, we will focus on systolic BP reduction and will recruit stroke patients with systolic BP ≥140 and <200 mmHg. Intravenous thrombolytic therapy, such as intravenous recombinant tissue plasminogen activator (rtPA), is rare in Chinese patients (1.6%).[22] Because of different BP lowering requirements in patients with intravenous rtPA, we will exclude them from the CATIS trial. Patients who have received or plan to receive endovascular thrombectomy will be excluded too.[23,24]

D2. Recruitment

We plan to recruit 4,776 eligible patients (2,388 for each randomization group) with acute ischemic stroke from 100 participating hospitals within the China Stroke Clinical Research Network. Each participating hospital will recruit approximately 50 patients. The recruitment period will last for two years. We have full support from the network.

The China Stroke Clinical Research Network (CSCRN) was established in 2008 and is supported by the National Clinical Research Center for Neurological Diseases. The network, which is led by Beijing Tiantan Hospital, consists in total of >390 hospitals in 30 provinces nationwide. The investigators and research staff at each clinical site of the network have been trained and certified in conducting clinical research in accordance with the principles of Good Clinical Practice and the use of neurological scoring systems. All network clinical sites have actively participated in large-scale multicenter clinical studies, including the China National Stroke Registry,[22] the CHANCE trial,[4] the SMART trial (Standard Medical Management in Secondary Prevention of Ischemic Stroke in China),[25] the GOLDEN BRIDGE-Acute Ischemic Stroke trial,[26] and other trials.

The study coordinators or study physicians at each hospital will approach all acute stroke patients as soon as allowed based on the patient's clinical condition (within 6 hours of hospitalization and within 48 hours of disease onset). Three BP measurements will be obtained objectively using an automated measurement device (Omron HBP-1300 Professional Blood Pressure Monitor), and one of four cuff sizes (pediatric, regular adult, large, or thigh) will be chosen on the basis of each participant's arm circumference. The mean of the three BP measures

Table 2. Eligibility criteria

Inclusion criteria

- Men and women aged ≥40 years
- Acute ischemic stroke confirmed by computed tomography (CT) or magnetic resonance imaging (MRI) of the head
- Stroke onset within 24-48 hours*
- Systolic blood pressure between 140 and <200 mmHg and diastolic blood pressure between 80-120 mmHg

Exclusion criteria

- Hemorrhagic stroke confirmed by CT or MRI of the head
- CT or MRI-diagnosed vascular malformation, tumor, abscess, or other major non-ischemic brain disease (e.g., multiple sclerosis)
- Extracranial or intracranial artery stenosis (≥70%) in both sides or the affected side based on imagine study
- Stroke caused by arteritis, migraine, vasospasm, or substance abuse
- Severe stroke (NIHSS score of ≥21)
- Coma (Glasgow Coma Scale [GCS] score <8)
- Preceding moderate or severe dependency (modified Rankin scale [mRS] score 3–5)
- Planned or probable revascularization (any angioplasty or vascular surgery) within 3 months after screening
- Intravenous thrombolytic therapy (such as intravenous rtPA) or mechanical thrombectomy
- Severe heart failure (NY Heart Association class III and IV) or left ventricular ejection fraction <35%
- Myocardial infarction or unstable angina within past 3 months
- History of atrial fibrillation.
- History of aortic dissection
- History of all-cause dementia
- Difficult-to-control hypertension (systolic blood pressure ≥170 mmHg despite use of ≥4 antihypertensive medications for ≥6 months)
- Acute renal failure or dialysis or estimated glomerular filtration rate (eGFR) <20 ml/min/1.73 m^2
- Any clinical conditions judged by the clinic team to likely limit the adherence to study procedures
- Inability to understand and/or follow research procedures due to mental, cognitive, or emotional disorders
- Unable to participate in the follow-up examination (e.g., cannot travel to the participating hospital)
- Participation in another clinical trial within 30 days
- Pregnant, currently trying to become pregnant, or of child-bearing potential and not using birth control
- Failure to obtain informed consent from a participant

* If stroke onset within 24 hours, patients will be asked to discontinue all home antihypertensive medications and will have their eligibility re-evaluated at 24 hours prior to randomization.

will be used to determine eligibility. Other criteria will be assessed by study questionnaires, clinical examinations, and laboratory tests (CT/MRI). Patients who meet other eligibility criteria, but for whom time from stroke onset is <24 hours, will be asked to discontinue home antihypertensive medications. Their eligibility will be re-evaluated at 24 hours prior to randomization.

E. Antihypertensive Treatment

All home BP medications will be discontinued at the screening/baseline examination among all eligible trial participants. The early treatment group will receive antihypertensive medications immediately after randomization

according to the study protocol, and the delayed treatment group will receive standard care according to the ASA clinical guidelines without antihypertensive treatment for the first seven days. After that, both groups will receive the same antihypertensive treatment with a systolic BP goal of <140 mmHg and diastolic BP goal of <90 mmHg.

E1. Blood pressure treatment goals and timeline

E1.1. Early antihypertensive treatment group

BP-lowering treatment will start immediately after randomization in the early antihypertensive treatment group. The target treatment goals are:

- Step 1 (within 24 hours after randomization): To lower systolic BP by 10-20% with an average of 15% (but systolic BP not lower than 130 mmHg and diastolic BP not lower than 80 mmHg) within the first 24 hours after randomization based on the participant's admission BP levels.
- Step 2 (within 7 days after randomization): To achieve systolic BP below 140 mmHg (but not lower than 120 mmHg) and diastolic BP below 90 mmHg (but not lower than 70 mmHg) and maintain this BP level afterward.

E1.2. Delayed antihypertensive treatment group

All home antihypertensive medications will be discontinued in the first seven days after randomization. Study participants will receive antihypertensive treatment on day eight after randomization and will target systolic BP <140 mmHg (but not lower than 120 mmHg) and diastolic BP <90 mmHg (but not lower than 70 mmHg) in two weeks. A BP check-up visit will be conducted in two weeks. If BP treatment goal is not achieved, antihypertensive medication will be titrated or a new medication will be added.

Except for the antihypertensive treatment, both randomization groups will receive the same standard care according to AHA/ASA and Chinese clinical guidelines for acute ischemic stroke management.[16,21]

E2. Antihypertensive agents

The CATIS-2 trial is designed to test BP reduction strategies — early antihypertensive treatment with lowering systolic BP by 10-20% within the first 24 hours after randomization and achieving systolic BP <140 and diastolic BP <90 mmHg within 7 days compared to delayed antihypertensive treatment until day eight — rather than test the efficacy of individual antihypertensive drugs. Several antihypertensive agents, including angiotensin-converting enzyme inhibitors (ACEIs), calcium channel blockers (CCBs), and diuretics will be used in the CATIS-2 trial. Based on experience from the CATIS trial, intravenous antihypertensive agents are preferable over oral agents because of their quick effect and easy administration to stroke patients.

A single optimal medication to lower BP in all patients with acute stroke has not been determined.[16] Based on the literature, ACEIs might provide better neurovascular protection,[27-29] and ACEIs could reduce BP after acute stroke without reducing cerebral blood flow.[29] Nicardipine IV has been recommended by AHA/ASA clinical guidelines, but it is difficult to administer without continuous BP monitoring.[16] In the CATIS-2 trial, Enalapri IV (ACEI) will be the first-line antihypertensive medication. CCBs and diuretics will be the second-line and third-line medications. In the early treatment group, oral antihypertensive medications (CCB as the first choice) should be added as soon as possible and replace Enalapri IV within the first week. Oral CCB will be given to patients in both the early and delayed treatment groups on day eight after randomization. The antihypertensive medications

and treatment goal (BP<140/90 mmHg) will be the same for the early treatment and delayed treatment groups beginning on day eight after stroke onset.

E3. Treatment algorithm in the early antihypertensive treatment group

All participants in the early BP-lowering treatment group will receive antihypertensive medications immediately after randomization in a staged sequence. Based on patients' baseline BP level, the first-line medication (ACEI IV) can be used alone, or in combination with second-line medication (CCBs), and third-line medication (diuretics) to achieve the target systolic BP lowering by 10% to 20% within the first 24 hours after randomization according to the treatment algorithm. The dosage and number of medications will be titrated to achieve systolic BP below 140 mmHg and diastolic BP below 90 mmHg within 7 days and maintain this BP level afterwards.

At any step above, if the BP target is achieved, medication will be continued at the same dosage to maintain the target BP level. If the BP target is not achieved by the above treatment algorithm, medications will be titrated up to the maximum dosage in the order of ACEIs, CCBs, and diuretics before adding another BP agent. A 4th agent can be added if needed. This treatment algorithm and these antihypertensive medications have been documented to be safe and effective in the CATIS trial.[11] Above treatment strategy are recommended but not exclusive, any individual antihypertensive therapy to achieve BP targets is allowed.

E4. Safety considerations

Study physicians will closely monitor stroke patients during the intervention. If systolic BP reduction is more than 20%, or systolic BP <130 mmHg, or diastolic BP <80 mmHg during the first 24 hours, we will titrate down or temporarily hold antihypertensive medications. Patients who experience severe conditions during the intervention, such as acute myocardial infarction and heart failure, will be withdrawn from the trial. Stroke patients with mean systolic BP ≥220 or diastolic BP ≥120 mmHg from three measurements at each of two time points apart 6 hours during intervention will be withdrawn from the trial. Study physicians will collect endpoint data for all withdrawn patients. These data will be used in statistical analysis according to the intention-to-treat principle. In addition, the DSMB will examine data every 6-12 months. If unexpected and highly significantly beneficial or harmful effects are identified, the DSMB could recommend stopping the trial. However, the DSMB's decision should not only depend on the statistical analysis alone. The overall benefits and harms to the study participants and the importance of the knowledge to be gained should be balanced.

F. Randomization and Masking

F1. Randomization

Randomization will be conducted centrally at the SDCC. The randomization will be stratified by participating hospitals. In each participating hospital, patients will be randomly assigned to the early antihypertensive treatment group or the delayed antihypertensive treatment group within each block. The block size will be random among 4, 6, and 8. The early BP lowering group will receive antihypertensive treatment immediately according to the study protocol, and the delayed BP lowering group will discontinue antihypertensive treatment for the first seven days after randomization.

The randomization schedules will be generated using SAS PROC PLAN in SAS and will be concealed at the

SDCC. When an eligible participant is ready for randomization, the site investigator/study coordinator will log-in to a password-protected website to obtain the randomization assignment.

F2. Masking

The study physicians and nurses will not be masked to patients' randomization assignments because of the different time points for initiating BP treatment between the two randomization groups. The data on BP will also be unmasked because they are management tools for attaining the specific treatment targets. However, the study neurologists and nurses who conduct the neurological and functional assessments and collect clinical outcome data at the baseline and follow-up examinations will be masked to patients' assignments. The members of the Outcome Adjudication Committee will also be masked to patients' randomization.

G. Study Visits and Data Collection

G1. Screening/baseline data collection

Data needed for the determination of patients' eligibility will be obtained at a screening/baseline examination which includes BP measurements, neurological and functional assessments, and CT/MRI scans immediately after hospital admission. Informed consent will be obtained at the baseline examination (**Table 3**). Information on medical history (including time from onset of stroke, history of hypertension, and use of antihypertensive medication) will be collected. A CT or MRI scan will be conducted to confirm the diagnosis of ischemic stroke. Three brachial BP measurements will be taken 30 seconds apart at the baseline examination with patients in the supine position. The neurological and functional assessments including the NIH Stroke Scale and the modified Rankin Scale will be performed. The modified Rankin Scale and NIHSS are widely used in general neurology practice in China and all study physicians have been trained in the use of these instruments. Clinical examination and routine laboratory tests will be conducted as part of patient care.

G2. Data collection during hospitalization

Based on experience from the CATIS trial, the median (interquartile range) of hospitalization duration in patients with acute ischemic stroke was 13.0 (9.0 to 14.0) days.[11] We will require all study participants to stay in

Table 3. Trial visit schedule and data collection

	Screening/ baseline	Time after randomization				BP visit in week 3	Follow-up visit in month 3
		Day 1 (first 24 hours)	Days 2-14	Day 8	Day 14/ Discharge		
Informed consent	X						
Medical history	X				X		X
CT or MRI	X						
Blood pressure	3 BPs	3 BPs/ every 3 hrs	3 BPs/ every 8 hrs		3 BPs	3 BPs	3 BPs
NIHSS and Rankin Scale	X				X		X
Clinical examination	X			X	X		X
Clinical laboratory measures	X				X		X
Antihypertensive treatment		start in intervention		start in control			
Death certificates					X		X

the hospital for seven to fourteen days during antihypertensive treatment in the proposed CATIS-2 trial. BP will be measured every 3 hours during the first 24 hours and every 8 hours during days 2-14. At each BP assessment, three brachial BP measurements will be taken 30 seconds apart using an automated measurement device (Omron HBP-1300 Professional Blood Pressure Monitor) with patients in the supine position. Neurological and functional assessments including the NIHSS and the modified Rankin Scale will be obtained 14 days or at discharge after the randomization. Clinical examinations and routine laboratory tests will be conducted as part of patient care. Death certificates and hospital data on causes of death will be obtained. All study outcomes will be collected by an observer who is blinded to the patient's randomization assignment.

G3. Blood pressure monitor visits

In week three after randomization (two weeks after initiation of antihypertensive medications in the delayed treatment group), those patients who are still alive at hospital discharge will be invited to come back for BP check-ups. If BP is uncontrolled (>140/90 mmHg), antihypertensive medication will be titrated or a new medication will be added.

G4. Data collection at 3-month follow-up visit

The trial participants will be contacted by telephone or in-person to set up a follow-up clinical visit in month 3 after randomization. Both groups will be assessed for study related outcomes in the same way and on the same schedule. Information on recurrent stroke, major vascular events, and deaths will be obtained from patients or their direct family members. Current treatment, including antihypertensive therapy, will also be recorded. Medical records will be collected for adjudication of study outcomes. Three BP readings will be obtained using a standard protocol. Neurological and functional assessments (modified Rankin Scale and NIHSS) will be performed. The follow-up data will be collected by an observer who is blinded to the patient's randomization assignment.

G5. Clinical laboratory measures

Serum lipid profiles (LDL, HDL, and triglycerides), fasting plasma glucose, and serum creatinine will be measured at the baseline examination and follow-up examinations at the local clinical laboratory as part of routine patient care. Chemistry profiles including electrolytes, blood urea nitrogen, and creatinine will be measured during hospitalization at the local clinical laboratory to monitor potential side effects.

G6. Measurement methods

G6.1. Blood pressure measurements

Three BP measurements will be obtained at the screening/baseline examination, every 3 hours in the first 24 hours and every 8 hours in days 2-14, and week 3 and month 3 after randomization by trained, certified nurses. BP will be measured according to a standard protocol recommended by the American Heart Association.[30] BP will be measured with the participant in a supine position after 5 minutes of quiet rest. In addition, participants will be advised to avoid alcohol, cigarettes, coffee/tea, and exercise for at least 30 minutes before their BP measurement. BP measurements will be obtained objectively using an automated measurement device (Omron HBP-1300 Professional Blood Pressure Monitor), and one of four cuff sizes (pediatric, regular adult, large, or thigh) will be chosen on the basis of each participant’s arm circumference. The need for a standardized protocol, not discarding any data, and careful transcription of all BP data into the study database will be enforced.

G6.2. NIH Stroke Scale (NIHSS) and modified Ranked Scale

Stroke severity will be assessed by trained neurologists using the NIHSS (scores range from 0 to 42, with higher scores indicating more severe neurologic deficits: 0=no stroke symptoms; 1-4=minor stroke; 5-15=moderate stroke; 16-20=moderate to severe stroke; and 21-42=severe stroke) at baseline, 14 days/discharge, and 3 months after randomization.[31] The modified Rankin Scale will be assessed by trained neurologists at 14 days/discharge and 3 months after randomization.[32] Scores on the modified Rankin Scale range from 0 to 6 with a score of 0 indicating no symptoms; a score of 5 indicating severe disability (i.e., bedridden, incontinent or requiring constant nursing care and attention); and a score of 6 indicating death. The NIHSS and the modified Rankin Scale have been translated into Chinese and are widely used in clinical practice and research in China.[4,11]

G6.3. Clinical outcome ascertainment

Clinical events occurring during follow-up will be ascertained equally in both treatment groups through self-reported events and medical record data collected by the study staff. Recurrent stroke, vascular events, and other clinical outcomes will be assessed using a standard medical event questionnaire. The hospital records, including medical history, findings on physical examination, laboratory findings, and discharge diagnosis will be abstracted by trained staff using a standard form. All deaths identified during follow-up will be verified by death certificates. An Outcome Adjudication Committee whose members are blinded to the patient's randomization assignment will review all primary and secondary outcomes. At least two committee members will review the medical records independently. The adjudication of recurrent stroke and other vascular events will be based on criteria established in the SPRINT trial.[33]

H. Study Endpoints

The primary hypothesis of the CATIS-2 trial is whether early antihypertensive treatment starting between the first 24-48 hours after the onset of an acute minor ischemic stroke will reduce the risk of a composite outcome of death and major disability over three months compared to delayed antihypertensive treatment for the first seven days.

H1. Primary endpoint

The primary endpoint for the CATIS-2 trial is a composite outcome of death and major disability (modified Rankin score ≥3) at 3 months.

H2. Major secondary endpoint

The major secondary endpoint for the CATIS trial is the first recurrent stroke event (hemorrhagic or ischemic) over three months of follow-up. Ischemic stroke is defined as an acute focal infarction of the brain or retina with one of the following: sudden onset of a new focal neurologic deficit, with clinical or imaging evidence of infarction lasting 24 hours or more and not attributable to a non-ischemic cause (i.e., not associated with brain infection, trauma, tumor, seizure, severe metabolic disease, or degenerative neurologic disease); a new focal neurologic deficit lasting for less than 24 hours and not attributable to a non-ischemic cause but accompanied by neuroimaging evidence of new brain infarction; or rapid worsening of an existing focal neurologic deficit lasting more than 24 hours and not attributable to a non-ischemic cause, accompanied by new ischemic changes on MRI or CT of the brain and clearly distinct from the index ischemic event. Hemorrhagic stroke was defined as acute extravasation of blood into the brain parenchyma or subarachnoid space with associated neurologic symptoms.[4,34] Stroke will be confirmed based on all available data, including symptoms and signs, imaging of the brain and

large vessels, and cardiac testing, e.g., echocardiography. Adjudicators will use their clinical judgment based on the available evidence to classify each case, and will be guided by pre-specified definitions and operational rules. Stroke will be classified as ischemic stroke, subarachnoid hemorrhage, intracerebral hemorrhage, other hemorrhage, other type, or unknown type. Ischemic stroke will be further sub-typed using the Causative Classification of Stroke system as evident, probable, or possible cases of large artery atherosclerosis, cardio-aortic embolism, small artery occlusion, other causes, and undetermined causes.[35]

H3. Other secondary endpoints

The other secondary endpoints for the CATIS II trial include important clinical outcomes at 3 months.

- Ordered 7-level categorical score of the modified Rankin Scale[36,37]
- All-cause mortality
- Major vascular disease events (vascular deaths, non-fatal stroke, non-fatal myocardial infarction, coronary revascularization, hospitalized or treated angina, and hospitalized or treated congestive heart failure)

I. Statistical Power and Data Analysis

I1. Sample size and study power

The sample size calculation is based on the primary outcome (a composite outcome of death and major disability). We calculated sample size based on the following assumptions:

- Significance level of 0.05 for a two-sided test
- Statistical power of 85%
- 3-month event rate of primary outcome of 25% in control group based on data from the CATIS trial[11]
- Proportional risk reduction of 15% (Rate ratio = 0.85)
- Proportion of loss to follow-up of 5% over 3 months

We estimated that 4,776 eligible patients (2,388 for each randomization group) are required based on a Likelihood Ratio test.[38,39] Power analysis was implemented in the Power Analysis and Sample Size software (NCSS, Kaysville, UT).

I2. Data management

All study data will be entered through a web-based data management system (DMS) first at local hospitals and then sent to Beijing Tiantan Hospital where the second entry will be conducted. The two independent databases will be sent to Tulane University for final data check and quality control. This web-based DMS is password-protected and designed to provide different levels of access to staff members at participating hospitals and at the SDCC. The SDCC staff will have additional functionality within the DMS to review study data, conduct quality control, generate queries, correct data, and create reports.

I3. Data analyses

Data will be analyzed according to participants' randomized treatment assignments regardless of their subsequent medications (intent-to-treat analysis). Two-sided P-values will be calculated and the significance level will be assessed at 0.05.

For the primary hypothesis, the difference in the proportion of a composite outcome of all-cause mortality and major disability at 3 months between the early treatment and delayed treatment groups will be compared using χ^2

tests. Relative risk regression will be used to obtain RRs and 95% CIs associated with early treatment.[40] Specifically, the probability of primary outcome will be modeled as a function of treatment assignment using a generalized linear model with log link and binomial error distribution. In cases in which the model fails to converge with the binomial error, we will substitute Gaussian error and use robust standard error estimates. We will use relative risk regression rather than logistic regression because the outcome is not rare, so the odds ratio is an overestimate of the relative risk. In addition, important co-variables, including baseline age, sex, systolic BP, NIHSS score, time from stroke onset to randomization, history of hypertension, and use of antihypertensive medication will be adjusted in a sensitivity analysis. We will use the same statistical analysis methods for secondary endpoints of all-cause mortality and major vascular disease events.

For major secondary hypothesis, time-to-event analysis methods will be used to compare the cumulative hazards of recurrent stroke between the early antihypertensive treatment group and the delayed antihypertensive treatment group.[41,42] Kaplan-Meier curves on cumulative hazards by follow-up time will be examined and the group differences will be tested using the log-rank test.[43,44] Cox proportional hazards models, implemented in the SAS PHREG procedure, will be used to obtain hazard ratios and 95% CIs.[45] In addition, important co-variables will be adjusted in a sensitivity analysis. The proportional hazards assumption will be assessed by examining log-log plots (the log of cumulative hazards vs. the log of follow-up time) and testing interactions. A significant test indicates the lack of proportionality.[46,47] If the proportional hazards assumption is violated, relative risk regression will be used to adjust for potential confounders.[40]

Neurological functional status measured by the modified Rankin scale at 3 months will be analyzed as ordinal categorical variables to improve statistical efficiency.[48] The median and inter-quartile range of modified Rankin scores will be calculated for the two comparison groups, and the difference will be tested using a Wilcoxon rank-sum test.[49] Ordinal logistic regression will be used to estimate the effect of BP reduction in the full range of the modified Rankin Scale.[50]

The average MoCA scores (with a maximum score of 30) at month 3 will be compared between the early treatment and delayed treatment groups using a Wilcoxon rank-sum test because of potential outliers and lack of normality in the MoCA scores. In addition, regression models will be used to adjust for important co-variables. We will fit semi-parametric models to relax the normality assumption and obtain robust estimates.[51] Using a standardized scoring system, the raw scores from the SF-12 will be transformed into a scale from 0 to 100, with score 0 representing poor health and 100 representing good health in each dimension. Standardized SF-12 scores will be compared between the early treatment and delayed treatment groups using methods described above.

Missing data: Complete case analysis will be used for the primary analyses. Multiple imputation for missing data will be conducted using the Markov chain Monte Carlo method in a sensitivity analysis.

SAS (SAS Institute, Cary, NC) and STATA (Stata Corp, College Station, TX) software will be used for statistical analyses.

I4. Subgroup analysis

Analysis of the effects of early antihypertensive treatment on outcomes by pre-specified subgroups will be conducted.

- Age, <65 vs. ≥ 65 years
- Gender, men vs. women

- Systolic BP at baseline, <160, 160-179, and ≥180 mmHg
- NIHSS score at baseline, 0-4, 5-15, and ≥16
- History of hypertension, yes vs. no
- Use of antihypertensive medications at admission, yes vs. no
- Major subtypes of ischemic stroke:[52,53] 1) large-artery atherosclerosis, 2) cardioembolism, 3) small-vessel occlusion, 4) stroke of other determined etiology, and 5) stroke of undetermined etiology

I5. Interim analysis

Interim analysis will focus on patient recruitment, baseline comparability of treatment arms, achievement of treatment goals in the active arm, sample size assumptions with regard to event rates, loss to follow-up, adverse effects data, and effect of treatment on the primary and secondary study outcomes. Interim analyses will coincide with the DSMB meeting.

Trial stopping rules: We will use stochastic curtailment for monitoring treatment effects.[54,55] This method will take into account the increased type 1 error rate due to repeated statistical tests on the data.

J. Quality Assurance and Quality Control

J1. Training and certification

All study investigators and staff will be required to attend trial-wide or regional training sessions. The training sessions will include orientation to the study protocol and treatment program, BP measurements, neurological dysfunction assessments (NIHSS and modified Rankin Scale), and clinical data collection. All BP observers will participate in a special training session on the use of a standardized protocol for measurement of BP. Satisfactory performance during a written test on knowledge of preparing study participants for BP measurement, selecting correct cuff size, and using standard techniques for BP measurement during a standardized videotape examination and during concordant measurements of BP with an instructor will be required for certification as a BP observer.

J2. Site visits

The site visit efforts will be coordinated by investigators and staff at Beijing Tiantan Hospital and Soochow University. Investigators and staff from the SDCC will travel to each participating hospital to assess adherence of clinical staff to the study protocol and MOP. These visits will provide opportunities to discuss and evaluate solutions related to issues that are critical to the study and will be conducted at least once per year.

J3. Quality monitoring and reporting

A quality control (QC) subcommittee will review the QC data regularly, including timeliness and completeness of study visits and data collection, intervention protocol adherence, and data quality. Reports on data QC will be sent to each participating hospital.

K. Bibliography and References Cited

1. Zhou M, Wang H, Zhu J, Chen W, Wang L, Liu S, Li Y, Wang L, Liu Y, Yin P, Liu J, Yu S, Tan F, Barber RM, Coates MM, Dicker D, Fraser M, González-Medina D, Hamavid H, Hao Y, Hu G, Jiang G, Kan H, Lopez AD, Phillips MR, She J, Vos T, Wan X, Xu G, Yan LL, Yu C, Zhao Y, Zheng Y, Zou X, Naghavi M, Wang Y, Murray CJ, Yang G, Liang X. Cause-specific mortality for 240 causes in China during 1990-2013: a systematic subnational analysis for the Global Burden of Disease Study 2013. Lancet. 2016;387(10015):251-72.

2. GBD 2013 Mortality and Causes of Death Collaborators. Global, regional, and national age-sex specific all-cause and cause-specific mortality for 240 causes of death, 1990-2013: a systematic analysis for the Global Burden of Disease Study 2013. Lancet. 2015;385(9963):117-71.

3. Feigin VL, Krishnamurthi RV, Parmar P, Norrving B, Mensah GA, Bennett DA, Barker-Collo S, Moran AE, Sacco RL, Truelsen T, Davis S, Pandian JD, Naghavi M, Forouzanfar MH, Nguyen G, Johnson CO, Vos T, Meretoja A, Murray CJ, Roth GA; GBD 2013 Writing Group; GBD 2013 Stroke Panel Experts Group. Update on the Global Burden of Ischemic and Hemorrhagic Stroke in 1990-2013: The GBD 2013 Study. Neuroepidemiology. 2015;45(3):161-76.

4. Wang Y, Wang Y, Zhao X, Liu L, Wang D, Wang C, Wang C, Li H, Meng X, Cui L, Jia J, Dong Q, Xu A, Zeng J, Li Y, Wang Z, Xia H, Johnston SC; CHANCE Investigators. Clopidogrel with aspirin in acute minor stroke or transient ischemic attack. N Engl J Med. 2013;369(1):11-19.

5. Qureshi AI, Ezzeddine MA, Nasar A, Suri MF, Kirmani JF, Hussein HM, Divani AA, Reddi AS. Prevalence of elevated blood pressure in 563,704 adult patients with stroke presenting to the ED in the United States. Am J Emerg Med. 2007; 25(1):32-8.

6. Wang Y, Xu J, Zhao X, Wang D, Wang C, Liu L, Wang A, Meng X, Li H, Wang Y. Association of hypertension with stroke recurrence depends on ischemic stroke subtype. Stroke. 2013; 44(5):1232-7.

7. Qureshi AI. Acute hypertensive response in patients with stroke: pathophysiology and management. Circulation 2008; 118(2):176-87.

8. Carlberg B, Asplund K, Hägg E. Factors influencing admission blood pressure levels in patients with acute stroke. Stroke 1991; 22:527-30.

9. Sandset EC, Bath PM, Boysen G, Jatuzis D, Kõrv J, Lüders S, Murray GD, Richter PS, Roine RO, Terént A, Thijs V, Berge E; SCAST Study Group. The angiotensin-receptor blocker candesartan for treatment of acute stroke (SCAST): a randomised, placebo-controlled, double-blind trial. Lancet 2011; 377(9767):741-50.

10. Bath PM, Woodhouse L, Scutt P, Krishnan K, Wardlaw JM, Bereczki D, Sprigg N, Berge E, Beridze M, Caso V, Chen C, Christensen H, Collins R, El Etribi A, Laska AC, Lees KR, Ozturk S, Phillips S, Pocock S, de Silva HA, Szatmari S, Utton S. Efficacy of nitric oxide, with or without continuing antihypertensive treatment, for management of high blood pressure in acute stroke (ENOS): a partial-factorial randomised controlled trial. Lancet 2015; 385(9968):617-28.

11. He J, Zhang Y, Xu T, Zhao Q, Wang D, Chen CS, Tong W, Liu C, Xu T, Ju Z, Peng Y, Peng H, Li Q, Geng D, Zhang J, Li D, Zhang F, Guo L, Sun Y, Wang X, Cui Y, Li Y, Ma D, Yang G, Gao Y, Yuan X, Bazzano LA, Chen J; CATIS Investigators. Effects of immediate blood pressure reduction on death and major disability in patients with acute ischemic stroke: the CATIS randomized clinical trial. JAMA. 2014; 311(5):479-89.

12. Lee M, Ovbiagele B, Hong KS, Wu YL, Lee JE, Rao NM, Feng W, Saver JL. Effect of Blood Pressure Lowering in Early Ischemic Stroke: Meta-Analysis. Stroke. 2015;46(7):1883-9.
13. Rothwell PM. Blood pressure in acute stroke: which questions remain? Lancet. 2015;385(9968):582-5.
14. Global Burden of Disease Study 2013 Collaborators. Global, regional, and national incidence, prevalence, and years lived with disability for 301 acute and chronic diseases and injuries in 188 countries, 1990-2013: a systematic analysis for the Global Burden of Disease Study 2013. Lancet. 2015; 386(9995):743-800.
15. He J, Gu D, Wu X, Reynolds K, Duan X, Yao C, Wang J, Chen CS, Chen J, Wildman RP, Klag MJ, Whelton PK. Major causes of death among men and women in China. N Engl J Med. 2005; 353:1124-34.
16. Jauch EC, Saver JL, Adams HP Jr, Bruno A, Connors JJ, Demaerschalk BM, Khatri P, McMullan PW Jr, Qureshi AI, Rosenfield K, Scott PA, Summers DR, Wang DZ, Wintermark M, Yonas H; American Heart Association Stroke Council; Council on Cardiovascular Nursing; Council on Peripheral Vascular Disease; Council on Clinical Cardiology. Guidelines for the early management of patients with acute ischemic stroke: a guideline for healthcare professionals from the American Heart Association/American Stroke Association. Stroke. 2013;44(3):870-947.
17. Oliveira-Filho J, Silva SC, Trabuco CC, Pedreira BB, Sousa EU, Bacellar A. Detrimental effect of blood pressure reduction in the first 24 hours of acute stroke onset. Neurology. 2003;61(8):1047-51.
18. Hankey GJ. Lowering blood pressure in acute stroke: the SCAST trial. Lancet. 2011; 377(9767):696-8.
19. Jordan JD, Powers WJ. Cerebral autoregulation and acute ischemic stroke. Am J Hypertens. 2012;25(9): 946-50.
20. Saver JL. Blood pressure management in early ischemic stroke. JAMA. 2014;311(5):469-70.
21. China National Guidelines for Prevention and Treatment of Cerebrovascular Diseases Committee. China National Guidelines for Prevention and Treatment of Cerebrovascular Diseases. People's Medical Publishing House: Beijing. 2005.
22. Wang Y, Liao X, Zhao X, Wang DZ, Wang C, Nguyen-Huynh MN, Zhou Y, Liu L, Wang X, Liu G, Li H, Wang Y; China National Stroke Registry Investigators. Using recombinant tissue plasminogen activator to treat acute ischemic stroke in China: analysis of the results from the Chinese National Stroke Registry (CNSR). Stroke. 2011;42(6):1658-64.
23. Powers WJ, Derdeyn CP, Biller J, Coffey CS, Hoh BL, Jauch EC, Johnston KC, Johnston SC, Khalessi AA, Kidwell CS, Meschia JF, Ovbiagele B, Yavagal DR; American Heart Association Stroke Council. 2015 American Heart Association/American Stroke Association Focused Update of the 2013 Guidelines for the Early Management of Patients With Acute Ischemic Stroke Regarding Endovascular Treatment: A Guideline for Healthcare Professionals From the American Heart Association/American Stroke Association. Stroke. 2015;46(10):3020-35.
24. Badhiwala JH, Nassiri F, Alhazzani W, Selim MH, Farrokhyar F, Spears J, Kulkarni AV, Singh S, Alqahtani A, Rochwerg B, Alshahrani M, Murty NK, Alhazzani A, Yarascavitch B, Reddy K, Zaidat OO, Almenawer SA. Endovascular Thrombectomy for Acute Ischemic Stroke: A Meta-analysis. JAMA. 2015;314(17):1832-43.

25. Peng B, Ni J, Anderson CS, Zhu Y, Wang Y, Pu C, Wu J, Wang J, Zhou L, Yao M, He J, Shan G, Gao S, Xu W, Cui L; SMART Investigators. Implementation of a structured guideline-based program for the secondary prevention of ischemic stroke in China. Stroke. 2014;45(2):515-9.
26. Wang Y, Li Z, Xian Y, Zhao X, Li H, Shen H, Wang C, Liu L, Wang C, Pan Y, Wang D, Prvu Bettger J, Fonarow GC, Schwamm LH, Smith SC Jr, Peterson ED, Wang Y; GOLDEN BRIDGE–AIS investigators. Rationale and design of a cluster-randomized multifaceted intervention trial to improve stroke care quality in China: The GOLDEN BRIDGE-Acute Ischemic Stroke. Am Heart J. 2015;169(6):767-774.
27. Schrader J, Luders S, Kulschewski A, Berger J, Zidek W, Treib J, Einhaupl K, Diener HC, Dominiak P. The ACCESS study: evaluation of acute candesartan cilexetil therapy in stroke survivors. Stroke. 2003; 34: 1699-703.
28. Potter JF, Robinson TG, Ford GA, Mistri A, James M, Chernova J, Jagger C. Controlling hypertension and hypotension immediately post-stroke (CHHIPS): a randomised, placebo-controlled, double-blind pilot trial. Lancet Neurol 2009; 8:48-56.
29. Elewa HF, Kozaka A, Johnson MH, Ergula A, Fagan SC. Blood pressure lowering after experimental cerebral ischemia provides neurovascular protection. J Hypertens. 2007; 25:855-9.
30. Pickering TG, Hall JE, Appel LJ, Falkner BE, Graves J, Hill MN, et al. Recommendations for blood pressure measurement in humans and experimental animals: Part 1: Blood pressure measurement in humans: A statement for professionals from the subcommittee of professional and public education of the American Heart Association Council on High Blood Pressure Research. Circulation. 2005;111:697-716.
31. Brott T, Adams HP Jr, Olinger CP, Marler JR, Barsan WG, Biller J, Spilker J, Holleran R, Eberle R, Hertzberg V. Measurements of acute cerebral infarction: a clinical examination scale. Stroke 1989; 20(7):864-70.
32. Bonita R, Beaglehole R. Modification of Rankin scale: recovery of motor function after stroke. Stroke 1988; 19:1497-500.
33. The SPRINT Study Research Group. The design and rationale of a multi-center clinical trial comparing two strategies for control of systolic blood pressure: The Systolic Blood Pressure Intervention Trial (SPRINT). Clin Trials. 2014;11(5):532–546.
34. World Health Organization, 1978 Cerebrovascular Disorders. Geneva: World Health Organization. ISBN 9241700432.
35. Ay H, Benner T, Arsava EM, Furie KL, Singhal AB, Jensen MB, Ayata C, Towfighi A, Smith EE, Chong JY, Koroshetz WJ, Sorensen AG. A computerized algorithm for etiologic classification of ischemic stroke - The causative classification of stroke system: Stroke. 2007;38:2979-2984.
36. Bath PM, Lees KR, Schellinger PD, Altman H, Bland M, Hogg C, Howard G, Saver JL; European Stroke Organisation Outcomes Working Group. Statistical analysis of the primary outcome in acute stroke trials. Stroke. 2012;43(4):1171-8.
37. Lees KR, Bath PM, Schellinger PD, Kerr DM, Fulton R, Hacke W, Matchar D, Sehra R, Toni D; European Stroke Organization Outcomes Working Group. Contemporary outcome measures in acute stroke research: choice of primary outcome measure. Stroke. 2012; 43(4):1163-70.

38. Fleiss JL., Levin B, Paik MC. Statistical Methods for Rates and Proportions. Third Edition. John Wiley & Sons. New York: 2003.
39. Machin D, Campbell M, Fayers P, Pinol A. Sample Size Tables for Clinical Studies, 2nd Edition. Blackwell Science. Malden, MA: 1997.
40. Moolgavkar SH, Venzon DJ. General relative risk regression models for epidemiologic studies. Am J Epidemiol. 1987;126:949-61.
41. Klein JP, Moeschberger ML. Survival analysis: techniques for censored and truncated regression. New York, NY: Springer-Verlag; 1997.
42. Hollander M, Wolfe DA. Nonparametric statistical methods (2nd Edition). New York: John Wiley & Sons. 1999.
43. Kaplan EL, Meier P. Nonparametric estimation from incomplete observations. J Am Stat Assoc. 1958;53:457-81.
44. Peto R, Peto J. Asymptotically efficient rank invariant test procedures. J Royal Stat Soci, Series A. 1972;35 (2):185-207.
45. Cox DR. Regression models and life tables (with discussion). J Royal Stat Soci, Series B, 1972;34:187-220.
46. Hess KR. Graphical methods for assessing violations of the proportional hazards assumption in Cox regression. Stat Med 1995; 14:1707-23.
47. Ng'Andu NH. An empirical comparison of statistical tests for assessing the proportional hazards assumption of Cox's model. Stat Med 1997; 16:611-26.
48. The Optimising Analysis of Stroke Trials (OAST) Collaboration. Can we improve the statistical analysis of stroke trials? Statistical reanalysis of functional outcomes in stroke trials. Stroke. 2007; 38:1911-15.
49. Wilcoxon F. Individual comparisons by ranking methods. Biomed Bull. 1945;1:80-83.
50. McCullagh P. Regression models for ordinal data (with discussion). J R Stat Soc B. 1980;42:109-142.
51. Tang W, He H, Tu XM. Applied categorical and count data analysis. Chapman and Hall/CRC: New York. 2012.
52. Adams HP Jr, Bendixen BH, Kappelle LJ, Biller J, Love BB, Gordon DL, Marsh EE 3rd. Classification of subtype of acute ischemic stroke. Definitions for use in a multicenter clinical trial. TOAST. Trial of Org 10172 in Acute Stroke Treatment. Stroke. 1993; 24:35-41.
53. Ay H, Benner T, Arsava EM, Furie KL, Singhal AB, Jensen MB, Ayata C, Towfighi A, Smith EE, Chong JY, Koroshetz WJ, Sorensen AG. A computerized algorithm for etiologic classification of ischemic stroke: the Causative Classification of Stroke System. Stroke. 2007; 38:2979-84.
54. Lan KKG, Wittes J. The B-value: A tool for monitoring data. Biometrics 1988; 44:579-85.
55. Davis BR, Hardy RJ. Upper bounds for type I and type II error rates in conditional power calculations. Communications in Statistics 1990; A19:3571-84.
