# Supplementary material for: Early vs Delayed Antihypertensive Treatment in Acute Single Subcortical Infarction: A Secondary Analysis of the CATIS-2 Randomized Clinical Trial
Source: JAMA Netw Open. 2024 Aug 30;7(8):e2430820. doi: 10.1001/jamanetworkopen.2024.30820 (PMC11365005; doi:10.1001/jamanetworkopen.2024.30820)
Supplement: Supplement 2. — Statistical Analysis Plan [file jamanetwopen-e2430820-s002.pdf]

China Antihypertensive Trial in Acute Ischemic Stroke II (CATIS-2)

Statistical Analysis Plan

Prepared by

Yuesong Pan, PhD

Aoming Jin, PhD

Mengxing Wang, MD

Hongyi Yan, MD

Beijing Tiantan Hospital, Capital Medical University, Beijing, China

Version 1.0

June 29, 2017

Table of Contents

1. Introduction....3
2. Study Objective....3
3. Study Endpoints....3
4. Statistical Hypotheses....3
5. Study Design....4
6. Sample Size Estimates....4
7. Analysis Populations....5
8. Treatment Comparisons....5
9. General Considerations for Data Analyses....5
10. Withdrawal and Missing Data....6
11. Study Population....6
12. Efficacy Analysis....7
13. Safety Analyses....7
14. Reference....8

1. Introduction

This statistical analysis plan (SAP) documents the planned statistical analyses for the CATIS-2 study and is based on the protocol, together with any subsequent amendments. This SAP is intended for the use of project team members and should be read in conjunction with the aforementioned protocol.

2. Study Objective

The primary objective of the study is to test whether early antihypertensive treatment starting between the first 24-48 hours after the onset of an acute ischemic stroke will reduce the risk of composite case-fatality and major disability (modified Rankin scale score≥3) at three months compared to delayed antihypertensive treatment (starting on day 8 after stroke onset).

The secondary objectives of the study are:

- To evaluate the differences in the risk of the first recurrent stroke (hemorrhagic or ischemic) within three months in the two groups.
- To evaluate the differences in the risk of the following endpoints at three months: ordered 7-level categorical score of the modified Rankin Scale, all-cause mortality, and major vascular events at three months in the two groups.

3. Study Endpoints

Primary endpoint: The primary endpoint for the CATIS-2 trial is a composite outcome of death and major disability (modified Rankin score ≥3) at 90 days.

Secondary endpoints

- The first recurrent stroke event (hemorrhagic or ischemic) over 90 days of follow-up.
- Ordered 7-level categorical score of the modified Rankin Scale at 90 days.
- All-cause mortality over 90 days of follow-up.
- Major vascular disease events (vascular deaths, non-fatal stroke, non-fatal myocardial infarction, coronary revascularization, hospitalized or treated angina, and hospitalized or treated congestive heart failure) over 90 days of follow-up.
- BP and use of antihypertensive medications

Safety endpoints

- Adverse events or serious adverse events within 3 months
- Severe conditions during the intervention, such as acute myocardial infarction and heart failure.

4. Statistical Hypotheses

The primary endpoint for this study is a composite outcome of death and major disability (modified Rankin score ≥3) at 90 days. The null hypothesis is that the proportion of a composite outcome of all-

cause mortality and major disability at 90 days in the early treatment and delayed treatment groups is the same. It will be tested using a two-sided significance level of 0.05.

H0: $\pi_1 = \pi_2$

H1: $\pi_1 \neq \pi_2$

where, π_1 is the proportion of a composite outcome of all-cause mortality and major disability at 90 days in the early treatment group; π_2 is the proportion of a composite outcome of all-cause mortality and major disability at 90 days in the delayed treatment group.

5. Study Design

The CATIS-2 is a multicenter, randomized, open-label, blinded-endpoints trial that will be conducted in 100 hospitals within the China Stroke Clinical Research Network. The CATIS-2 trial is designed to test whether early antihypertensive treatment starting between the first 24-48 hours after the onset of an acute ischemic stroke, compared to delayed antihypertensive treatment for the first seven days, will reduce the risk of mortality and major disability events, recurrent stroke, and cardiovascular disease (CVD) over three months. The primary study endpoint is the composite outcome of death and major disability within 90 days after randomization. The secondary endpoints are the first recurrent stroke (hemorrhagic or ischemic), modified Rankin score, all-cause mortality, and major vascular events over three months. The CATIS-2 trial will recruit 4,776 eligible patients (2,388 for each group) aged ≥22 years with acute ischemic stroke confirmed by CT or MRI onset between 24-48 hours and with systolic BP between 140 and <220 mmHg from 100 hospitals and randomly assign them to two parallel arms:

Planned Analyses

The analyses that are detailed in this SAP will be performed only when the database has been locked, all protocol violators identified, and treatment allocations have been unblinded. Membership of the Full Analysis and Per Protocol populations will be determined using the rules set out in this SAP. At a date to be agreed within the project team, a data look will be performed. This will involve production of all data displays on a subset of the data using dummy treatment codes. These are produced purely as an aide to the pre-programming of the study and no unblinding will occur.

Interim Analyses

Interim analysis is not planned for this trial. An independent data and safety monitoring board (DSMB) monitored the trial's progress and periodically will review the safety and efficacy data, focusing on patient recruitment, baseline comparability of treatment arms, achievement of treatment goals in the active arm, sample size assumptions with regard to event rates, loss to follow-up, and adverse effects data. Interim analysis will be arranged if DSMB considered it is necessary. This will take into account the increased type 1 error rate due to repeated statistical tests on the data.

6. Sample Size Estimates

The sample size calculation is based on the primary outcome (a composite outcome of death and major disability). We calculated sample size based on the following assumptions:

- Significance level of 0.05 for a two-sided test
- Statistical power of 85%
- 3-month event rate of primary outcome of 25% in control group based on data from the CATIS trial
- Proportional risk reduction of 15% (rate ratio = 0.85)
- Proportion of loss to follow-up of 5% over 3 months

We estimated that 4,776 eligible patients (2,388 for each randomization group) are required based on a Likelihood Ratio test. Power analysis was implemented in the Power Analysis and Sample Size software (NCSS, Kaysville, UT).

7. Analysis Populations

Intention-to-treat analyses will be conducted, in which study outcomes will be compared between patients according to their randomization assignment, regardless of their actual adherence to the intervention.

8. Treatment Comparisons

The CARSI-2 will compare the early antihypertensive treatment starting between the first 24-48 hours after the onset of an acute ischemic stroke versus delayed antihypertensive treatment (starting on day 8 after stroke onset) on the risk of composite case-fatality and major disability (modified Rankin scale score≥3) at 90 days.

9. General Considerations for Data Analyses

All statistical analyses will be performed using SAS 9.4 (SAS Institute, Cary, NC). Effect sizes will be presented as the point estimates and 95% confidence intervals.

Subgroup analysis

The subgroup analysis of the effects of early antihypertensive treatment vs. delayed antihypertensive treatment on clinical outcomes will be conducted by pre-specified subgroups:

- Age, <65 vs. ≥65 years
- Sex, men vs. women
- Systolic BP at baseline, <160, 160-179, and ≥180 mmHg
- NIHSS score at baseline, 0-4, 5-15, and ≥16
- History of hypertension, yes vs. no
- Use of antihypertensive medications at admission, yes vs. no
- Major subtypes of ischemic stroke: 1) large-artery atherosclerosis, 2) cardioembolism, 3) small-vessel occlusion, 4) stroke of other determined etiology, and 5) stroke of undetermined etiology

Multiple comparisons and multiplicity

The critical values will not be adjusted for in the primary and secondary study outcomes. There will be one primary study outcome – a composite outcome of death and major disability (modified Rankin score ≥ 3) at 90 days.

The Bonferroni correction method will be used to adjust the critical value for interaction tests in the subgroup analyses. A 2-sided p-value 0.05/7 will be considered statistically significant in the subgroup analyses.

10. Withdrawal and Missing Data

If any subject withdraws prematurely from the study (prior to the final visit at 90 days), they are required to complete the withdrawal visit at the participating hospital clinics. The reasons for withdrawal will be presented in a summary table. Subjects who withdraw before the end of the study but participate some follow-up visits will be included in the analysis. Subjects who withdraw study consent will be excluded from analysis of any endpoint.

Complete case analysis will be used for the primary analyses. Multiple imputation for missing data will be conducted using the Markov chain Monte Carlo method in a sensitivity analysis.

11. Study Population

Disposition of participants

The number of patients in each analysis population will be presented, and the total number of pateints attending each clinic visit will also be summarized by treatment group.

The number of patients randomised, completed and prematurely withdrawn from the study will be presented for each treatment group. The primary reasons for withdrawal both prior to and post randomisation will also be presented.

A data display listing and summary of deviations from the inclusion/exclusion criteria will be presented for all patients who were either entered or randomized into the trial.

Demographic and baseline characteristics

The following demographic information will be listed and summarized for patients in each treatment group: baseline age, sex, systolic BP, NIHSS score, time from stroke onset to randomization, history of hypertension, and use of antihypertensive medication.

The continuous variables followed normal distribution will be presented as mean and standard deviation, and the continuous variable followed skewness distribution will be presented as median and interquartile range; categorical data will be presented as n (%). Analysis of variance or Wilcoxon rank sum test will be used for comparison of continuous data, and Chi-squared tests or Fisher exact test will be used for categorical data.

12. Efficacy Analysis

Primary efficacy analysis: The primary endpoint is a composite outcome of death and major disability (modified Rankin score ≥3) at 90 days. Data will be analyzed according to participants' randomized assignments regardless of their subsequent medications (intent-to-treat analysis). Two-sided p-value of 0.05 will be the significance level.

Logistic regression will be used to obtain odds ratios (ORs) and 95% CIs associated with the early treatment vs. delayed treatment. The odds of primary outcome will be modeled as a function of treatment assignment using a generalized linear model with log link and binomial error distribution. In addition, important co-variables, including baseline age, sex, systolic BP, NIHSS score, time from stroke onset to randomization, history of hypertension, and use of antihypertensive medication will be adjusted in a sensitivity analysis.

Subgroup analyses: Summary tables will be produced for the predefined subgroups and interactions between treatment and these subgroups will be investigated, using a logistic regression model. A separate model will be used for each interaction to determine its significance. This will also be presented graphically on a forest plot.

Secondary efficacy analyses: Logistic regression analysis will be used for binary secondary study outcomes, such as recurrent stroke, vascular events, and all-cause mortality (see primary efficacy analysis).

Ordered 7-level categorical score of the modified Rankin Scale: Neurological functional status measured by the modified Rankin scale at 14 and 90 days will be analyzed as ordinal categorical variables to improve statistical efficiency. The median and inter-quartile range of modified Rankin scores will be calculated for the two comparison groups, and the difference will be tested using a Wilcoxon rank-sum test. Ordinal logistic regression will be used to estimate the effect of BP reduction in the full range of the modified Rankin Scale.

13. Safety Analyses

Adverse events: Adverse events (AEs) will be coded using the MedDRA coding dictionary (Version 6.0 or a later release) and grouped by system organ class (as detailed in the study protocol). Within each treatment group, the number and percentage of subjects experiencing an AE will be summarized by system organ class and preferred term, and Chis-square test or Fisher's Exact test will be used to compare the number of patients with AE event between treatment groups.

Serious adverse events: Summary tables and data displays will be provided for serious adverse events. In addition, all deaths and serious AE's will be documented in a case narrative format in the clinical study report. The number of deaths occurring over the treatment period will be summarized, and Chis-square test or Fisher's Exact test will be used to compare the number of deaths between treatment groups.

14. Reference

1. Qureshi AI. Acute hypertensive response in patients with stroke: pathophysiology and management. Circulation 2008; 118(2):176-87.
2. He J, Zhang Y, Xu T, Zhao Q, Wang D, Chen CS, Tong W, Liu C, Xu T, Ju Z, Peng Y, Peng H, Li Q, Geng D, Zhang J, Li D, Zhang F, Guo L, Sun Y, Wang X, Cui Y, Li Y, Ma D, Yang G, Gao Y, Yuan X, Bazzano LA, Chen J; CATIS Investigators. Effects of immediate blood pressure reduction on death and major disability in patients with acute ischemic stroke: the CATIS randomized clinical trial. JAMA. 2014; 311(5):479-89.
