# Supplementary material for: Early vs Delayed Antihypertensive Treatment in Acute Single Subcortical Infarction: A Secondary Analysis of the CATIS-2 Randomized Clinical Trial
Source: JAMA Netw Open. 2024 Aug 30;7(8):e2430820. doi: 10.1001/jamanetworkopen.2024.30820 (PMC11365005; doi:10.1001/jamanetworkopen.2024.30820)
Supplement: Supplement 3. — eTable 1. Baseline Characteristics Between Patients With and Without Brain MRI Scans During Hospitalization eTable 2. Baseline Characteristics of the Included Patients According to 2 Treatment Arms eTable 3. Use of Antihypertensive Medications During Follow-Up in SSI Patients With and Without PAD Stenosis eTable 4. Clinical Outcomes Between Early and Delayed Antihypertensive Treatment Among Patients With SSI eTable 5. Clinical Outcomes Between Early and Delayed Antihypertensive Treatment Among Patients With SSI Stratified by Infarction Diameter eTable 6. The Sensitivity Analysis of 2 Treatment Arms on Clinical Outcomes in SSI Patients With and Without PAD Stenosis eTable 7. Early Versus Delayed Antihypertensive Treatment on Clinical Outcomes in Patients With PAD Stenosis Evaluated by MRA or CTA eFigure 1. Examples of Single Subcortical Infarction (SSI) With or Without Parent Artery Disease (PAD) Stenosis eFigure 2. Flowchart of the Current Study [file jamanetwopen-e2430820-s003.pdf]

Supplemental Online Content

Wei Y, Xie X, Pan Y, et al. Early vs delayed antihypertensive treatment in acute single subcortical infarction: a secondary analysis of the CATIS-2 randomized clinical trial. *JAMA Netw Open.* 2024;7(9):e2430820. doi:10.1001/jamanetworkopen.2024.30820

eTable 1. Baseline Characteristics Between Patients With and Without Brain MRI Scans during Hospitalization

eTable 2. Baseline Characteristics of the Included Patients According to 2 Treatment Arms

eTable 3. Use of Antihypertensive Medications during Follow-up in SSI Patients With and Without PAD Stenosis

eTable 4. Clinical Outcomes Between Early and Delayed Antihypertensive Treatment Among Patients With SSI

eTable 5. Clinical Outcomes Between Early and Delayed Antihypertensive Treatment Among Patients With SSI Stratified by Infarction Diameter

eTable 6. The Sensitivity Analysis of 2 Treatment Arms on Clinical Outcomes in SSI Patients With and Without PAD Stenosis

eTable 7. Early Versus Delayed Antihypertensive Treatment on Clinical Outcomes in Patients With PAD Stenosis Evaluated by MRA or CTA

eFigure 1. Examples of Single Subcortical Infarction (SSI) With or Without Parent Artery Disease (PAD) Stenosis

eFigure 2. Flowchart of the Current Study

This supplemental material has been provided by the authors to give readers additional information about their work.

© 2024 Wei Y et al. *JAMA Network Open.*

eTable 1. Baseline Characteristics Between Patients With and Without Brain MRI Scans during Hospitalization

Characteristics	Without MRI scans (N=1905)	With MRI scans (N=2897)	*P* value
Age, mean (SD), y	64.3 (10.5)	63.3 (10.1)	<.01
Sex, No. (%)			.73
Male	1233 (64.7)	1889 (65.2)	
Female	672 (35.3)	1008 (34.8)	
Blood pressure at entry, mean (SD), mm Hg			
Systolic	161.7 (15.0)	163.6 (14.8)	<.001
Diastolic	91.6 (10.2)	92.0 (9.8)	.26
Baseline NIHSS score, median (IQR)	3 (2-5)	3 (2-5)	.34
Medical history, No. (%)			
Hypertension	1545 (81.1)	2284 (78.8)	.06
Diabetes mellitus	457 (24.0)	709 (24.5)	.70
Dyslipidemia	49 (2.6)	100 (3.5)	.09
Previous stroke	506 (26.6)	730 (25.2)	.29
Previous TIA	7 (0.4)	13 (0.5)	.67
Coronary heart disease	139 (7.3)	242 (8.4)	.18
Current smoking, No. (%)	574 (30.1)	1070 (37.0)	<.001
Previous medications use, No. (%)			
Antihypertensive therapy	984 (51.7)	1556 (53.7)	.16
Antiplatelet therapy	172 (9.0)	315 (10.9)	.04
Lipid-lowering therapy	97 (5.1)	221 (7.6)	<.001
Etiology of stroke, No. (%)			<.001
Large-artery atherosclerosis	1198 (62.9)	1168 (40.3)	

© 2024 Wei Y et al. *JAMA Network Open.*

Small-artery disease	594 (31.2)	1477 (51.0)	
Others	113 (5.9)	252 (8.7)	
Time from onset to randomization, median (IQR), hours	35.0 (27.5-43.7)	36.9 (29.3-44.6)	<.001

Abbreviations: MRI, magnetic resonance imaging; SD, standard deviation; NIHSS, National Institutes of Health Stroke Scale; IQR, interquartile range; TIA, transient ischemic attack.

© 2024 Wei Y et al. *JAMA Network Open.*

eTable 2. Baseline Characteristics of the Included Patients According to 2 Treatment Arms

Characteristics	Early treatment (n=491)	Delayed treatment (n=506)
Age, mean (SD), y	62.7 (9.8)	62.0 (9.9)
Sex, No. (%)		
Male	308 (62.7)	304 (60.1)
Female	183(37.3)	202(39.9)
Blood pressure at entry, mean (SD), mm Hg		
Systolic	163.2 (14.4)	163.8 (14.7)
Diastolic	91.4 (9.3)	91.7 (9.9)
Baseline NIHSS score, median (IQR)	3 (2-5)	3 (2-5)
Medical history, No. (%)		
Hypertension	377 (76.8)	408 (80.6)
Diabetes mellitus	124 (25.3)	117 (23.1)
Dyslipidemia	13 (2.7)	17 (3.4)
Previous stroke	97 (19.8)	108 (21.3)
Previous TIA	3 (0.6)	5 (1.0)
Coronary heart disease	35 (7.1)	29 (5.7)
Current smoking, No. (%)	174 (35.4)	164 (32.5)
Previous medications use, No. (%)		
Antihypertensive therapy	240 (48.9)	289 (57.1)
Antiplatelet therapy	53 (10.8)	46 (9.1)
Lipid-lowering therapy	29 (5.9)	35 (6.9)
Time from onset to randomization, median (IQR), hours	37.4 (30.0-45.0)	37.7 (29.7-45.2)
Time from onset to imaging, median (IQR), hours	30.0(19.6-52.0)	30.5(21.0-54.1)

© 2024 Wei Y et al. *JAMA Network Open.*

Time from randomization to imaging, median (IQR), hours	-4.1(-19.1-16.0)	-3.4(-19.0-17.8)

Abbreviations: SD, standard deviation; NIHSS, National Institutes of Health Stroke Scale; IQR, interquartile range; TIA, transient ischemic attack.

© 2024 Wei Y et al. *JAMA Network Open.*

eTable 3. Use of Antihypertensive Medications during Follow-up in SSI Patients With and Without PAD Stenosis

	SSI+PAD		SSI-PAD	
	Early treatment, No. (%)	Delayed treatment, No. (%)	Early treatment, No. (%)	Delayed treatment, No. (%)
**Use of antihypertensive medications within 24 hours of randomization**				
Number of medications				
0	1 (1.6)	52 (100.0)	20 (4.7)	454 (100.0)
1	34 (53.1)	0	296 (69.3)	0
2	22 (34.4)	0	101 (23.7)	0
3	7 (10.9)	0	7 (1.6)	0
⩾4	0	0	3 (0.7)	0
Class of medications				
Angiotensin-converting-enzyme inhibitors	28 (43.8)	0	155 (36.3)	0
Angiotensin II receptor blockers	2 (3.1)	0	26 (6.1)	0
Diuretics	0	0	5 (1.2)	0
Calcium channel blockers	43 (67.2)	0	275 (64.4)	0
Beta blockers	1 (1.6)	0	2 (0.5)	0
Alpha blockers	1 (1.6)	0	3 (0.7)	0
Others	0	0	0	0
**Use of antihypertensive medications at day 14 or hospital discharge**				
Number of medications				
0	5 (7.8)	4 (7.7)	31 (7.3)	60 (13.3)
1	32 (50.0)	30 (57.7)	255 (59.7)	288 (63.6)
2	24 (37.5)	16 (30.8)	117 (27.4)	97 (21.4)

© 2024 Wei Y et al. *JAMA Network Open.*

3	3 (4.7)	2 (3.9)	24 (5.6)	7 (1.6)
⩾4	0	0	0	1 (0.2)
Class of medications				
Angiotensin-converting-enzyme inhibitors	17 (26.6)	10 (19.2)	90 (21.1)	77 (17.0)
Angiotensin II receptor blockers	3 (4.7)	3 (5.8)	36 (8.4)	26 (5.7)
Diuretics	0	1 (1.9)	9 (2.1)	6 (1.3)
Calcium channel blockers	49 (76.6)	43 (82.7)	323 (75.6)	324 (71.5)
Beta blockers	1 (1.6)	2 (3.9)	7 (1.6)	5 (1.1)
Alpha blockers	0	0	4 (0.9)	0
Others	1 (1.6)	0	3 (0.7)	0
**Use of antihypertensive medications at day 90**				
Number of medications				
0	8 (12.5)	3 (5.8)	48 (11.3)	66 (14.6)
1	25 (39.1)	30 (57.7)	244 (57.3)	272 (60.0)
2	22 (34.4)	14 (26.9)	103 (24.2)	86 (19.0)
3	8 (12.5)	5 (9.6)	30 (7.0)	28 (6.2)
⩾4	1 (1.6)	0	1 (0.2)	1 (0.2)
Class of medications				
Angiotensin-converting-enzyme inhibitors	19 (29.7)	13 (25.0)	97 (22.8)	86 (19.0)
Angiotensin II receptor blockers	12 (18.8)	7 (13.5)	76 (17.8)	63 (13.9)
Diuretics	6 (9.4)	4 (7.7)	25 (5.9)	24 (5.3)
Calcium channel blockers	50 (78.1)	43 (82.7)	298 (70.0)	318 (70.2)
Beta blockers	2 (3.1)	2 (3.9)	14 (3.3)	6 (1.3)

© 2024 Wei Y et al. *JAMA Network Open.*

Alpha blockers	0	0	5 (1.2)	4 (0.9)
Others	2 (3.1)	0	3 (0.7)	1 (0.2)

Abbreviations: SSI, single subcortical infarction; PAD, parent artery disease.

© 2024 Wei Y et al. *JAMA Network Open.*

eTable 4. Clinical Outcomes Between Early and Delayed Antihypertensive Treatment Among Patients With SSI

Outcomes	Early treatment, No./total No. (%)	Delayed treatment, No./total No. (%)	OR (95% CI)	*P* Value
Primary outcome				
Functional dependency or death at 90 days (mRS score ⩾3), No. (%)	43/490 (8.8)	36/505 (7.1)	1.25 (0.79-1.99)	.34
Secondary outcome				
Ordinal mRS scores at 90 days, No. (%)			1.01 (0.81-1.28)[a]	.91
0	183/490 (37.4)	186/505 (36.8)		
1	206/490 (42.0)	220/505 (43.6)		
2	58/490 (11.8)	63/505 (12.5)		
3	32/490 (6.5)	23/505 (4.6)		
4	10/490 (2.0)	10/505 (2.0)		
5	0	3/505 (0.6)		
6	1/490 (0.2)	0		
Recurrent stroke, No. (%)	31/491 (6.3)	26/506 (5.1)	1.24 (0.73-2.13)	.43
Major vascular events, No. (%)	31/491 (6.3)	29/506 (5.7)	1.11 (0.66-1.87)	.70
END, No. (%)	19/491 (3.9)	20/505 (4.0)	0.98 (0.51-1.85)	.94

Abbreviations: SSI, single subcortical infarction; OR, odds ratio; mRS, modified Rankin Scale; END, early neurological deterioration.

[a] Odds of a 1 unit higher modified Rankin Scale score.

© 2024 Wei Y et al. *JAMA Network Open.*

eTable 5. Clinical Outcomes Between Early and Delayed Antihypertensive Treatment Among Patients With SSI Stratified by Infarction Diameter

Outcomes	Median diameter of infarction, mm	Early treatment, No./total No. (%)	Delayed treatment, No./total No. (%)	OR (95% CI)	*P* Value	*P* Value for Interaction
Primary outcome						
Functional dependency or death at 90 days (mRS score ⩾3)	⩽20	31/446 (7.0)	25/460 (5.4)	1.30(0.76-2.24)	.34	.84
	>20	12/44 (27.3)	11/45 (24.4)	1.16(0.45-3.00)	.76	
Secondary outcome						
Ordinal mRS scores at 90 days, Median (IQR)	⩽20	1 (0-1)	1 (0-1)	0.99(0.78-1.27)[a]	.95	.49
	>20	1 (1-3)	1 (0-2)	1.19(0.57-2.51)[a]	.64	
Recurrent stroke	⩽20	27/447 (6.0)	22/461 (4.8)	1.28(0.72-2.29)	.40	.78
	>20	4/44 (9.1)	4/45 (8.9)	1.03(0.24-4.38)	.97	
Major vascular events	⩽20	27/447 (6.0)	25/461 (5.4)	1.12(0.64-1.96)	.69	.91
	>20	4/44 (9.1)	4/45 (8.9)	1.03(0.24-4.38)	.97	
END	⩽20	16/447 (3.6)	17/460 (3.7)	0.97(0.48-1.94)	.93	.95
	>20	3/44 (6.8)	3/45 (6.7)	1.02(0.20-5.37)	.98	

Abbreviations: SSI, single subcortical infarction; OR, odds ratio; mRS, modified Rankin Scale; END, early neurological deterioration.

[a] Odds of a 1 unit higher modified Rankin Scale score.

© 2024 Wei Y et al. *JAMA Network Open.*

eTable 6. The Sensitivity Analysis of 2 Treatment Arms on Clinical Outcomes in SSI Patients With and Without PAD Stenosis

Outcomes	SSI+PAD, No./total No. (%)				SSI-PAD, No./total No. (%)				*P* Value for Interaction[a]
	Early treatment	Delayed treatment	aOR (95% CI)[a]	*P* Value	Early treatment	Delayed treatment	aOR (95% CI)[a]	*P* Value	
Primary outcome									
Functional dependency or death at 90 days (mRS score⩾3)	15/64 (23.4)	4/52 (7.7)	4.78 (1.19-19.16)	.03	28/426 (6.6)	32/453 (7.1)	1.09 (0.61-1.92)	.78	.08
Secondary outcome									
Ordinal mRS scores at 90 days			2.72 (1.24-5.99)[b]	.01			0.91 (0.70-1.17)[b]	.45	.02
0	15/64 (23.4)	19/52 (36.5)			168/426 (39.4)	167/453 (36.9)			
1	25/64 (39.1)	24/52 (46.2)			181/426 (42.5)	196/453 (43.3)			
2	9/64 (14.1)	5/52 (9.6)			49/426 (11.5)	58/453 (12.8)			
3	10/64 (15.6)	2/52 (3.9)			22/426 (5.2)	21/453 (4.6)			
4	4/64 (6.3)	0			6/426 (1.4)	10/453 (2.2)			
5	0	2/52 (3.9)			0	1/453 (0.2)			
6	1/64 (1.6)	0			0	0			

© 2024 Wei Y et al. *JAMA Network Open.*

Recurrent stroke	6/64 (9.4)	3/52 (5.8)	2.52 (0.50-12.76)	.26	25/427 (5.9)	23/454 (5.1)	1.12 (0.62-2.02)	.72	.74
Major vascular events	6/64 (9.4)	3/52 (5.8)	2.52 (0.50-12.76)	.26	25/427 (5.9)	26/454 (5.7)	0.99 (0.56-1.76)	.97	.64
END	5/64 (7.8)	4/52 (7.7)	1.39 (0.29-6.73)	.69	14/427 (3.3)	16/453 (3.5)	0.84 (0.40-1.78)	.65	.89

Abbreviations: SSI, single subcortical infarction; PAD, parent artery disease; aOR, adjusted odds ratio; mRS, modified Rankin Scale; END, early neurological deterioration.

[a] Adjusted for age, sex, baseline NIHSS score, history of hypertension, history of diabetes mellitus, history of dyslipidemia, previous stroke, previous medication use of antihypertensive therapy, antiplatelet therapy and lipid-lowering therapy, and time from onset to imaging.

[b] Odds of a 1-unit higher modified Rankin Scale score.

© 2024 Wei Y et al. *JAMA Network Open.*

eTable 7. Early Versus Delayed Antihypertensive Treatment on Clinical Outcomes in Patients With PAD Stenosis Evaluated by MRA or CTA

Outcomes	SSI+PAD, No./total No. (%) (N=121)				SSI-PAD, No./total No. (%) (N=920)				*P* Value for Interaction
	Early treatment (n=68)	Delayed treatment (n=53)	OR (95% CI)	*P* Value	Early treatment (n=446)	Delayed treatment (n=474)	OR (95% CI)	*P* Value	
Primary outcome									
Functional dependency or death at 90 days (mRS score⩾3)	16/68 (23.5)	4/53 (7.6)	3.77 (1.18-12.06)	.03	29/445 (6.5)	33/473 (7.0)	0.93 (0.56-1.56)	.78	.03
Secondary outcome									
Ordinal mRS scores at 90 days			2.20 (1.12-4.32)[a]	.02			0.91 (0.71-1.15)[a]	.42	.01
0	17/68 (25.0)	19/53 (35.9)			176/445 (39.6)	176/473 (37.2)			
1	25/68 (36.8)	25/53 (47.2)			189/445 (42.5)	205/473 (43.3)			
2	10/68 (14.7)	5/53 (9.4)			51/445 (11.5)	59/473 (12.5)			
3	11/68 (16.2)	2/53 (3.8)			22/445 (4.9)	22/473 (4.7)			
4	4/68 (5.9)	0			6/445 (1.4)	10/473 (2.1)			
5	0	2/53 (3.8)			1/445 (0.2)	1/473 (0.2)			
6	1/68 (1.5)	0			0	0			

© 2024 Wei Y et al. *JAMA Network Open.*

Recurrent stroke	6/68 (8.8)	3/53 (5.7)	1.61 (0.38-6.77)	.51	27/446 (6.1)	25/474 (5.3)	1.16 (0.66-2.03)	.61	.67
Major vascular events	6/68 (8.8)	3/53 (5.7)	1.61 (0.38-6.77)	.51	27/446 (6.1)	28/474 (5.9)	1.03 (0.60-1.77)	.93	.56
END	5/68 (7.4)	4/53 (7.6)	0.97 (0.25-3.81)	.97	14/446 (3.1)	17/473 (3.6)	0.87 (0.42-1.79)	.70	.89

Abbreviations: MRA, magnetic resonance angiography; CTA, computed tomography angiography; SSI, single subcortical infarction; PAD, parent artery disease; OR, odds ratio; mRS, modified Rankin Scale; END, early neurological deterioration.

[a] Odds of a 1-unit higher modified Rankin Scale score.

© 2024 Wei Y et al. *JAMA Network Open.*

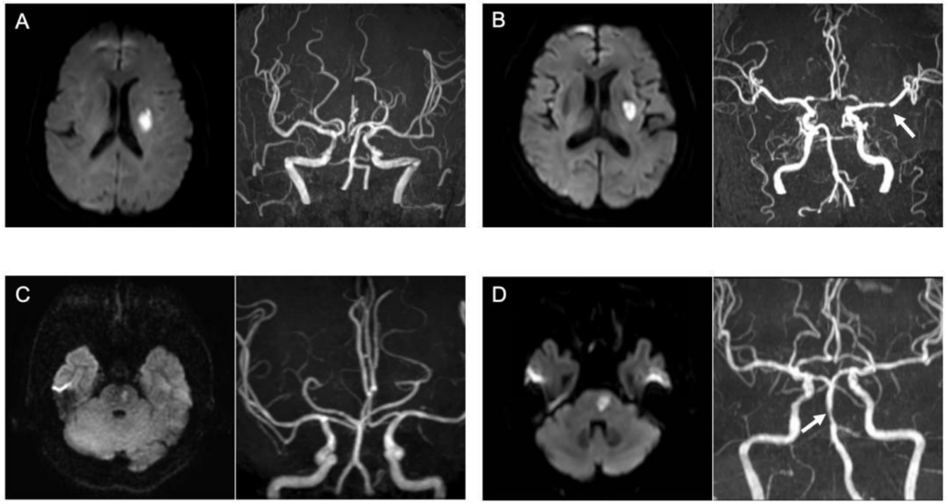

eFigure 1. Examples of Single Subcortical Infarction (SSI) With or Without Parent Artery Disease (PAD) Stenosis

(A) SSI without PAD stenosis in the territory of the middle cerebral artery; (B) SSI with PAD stenosis (arrow) in the territory of the middle cerebral artery; (C) SSI without PAD stenosis in the territory of the basilar artery; (D) SSI with PAD stenosis (arrow) in the territory of the basilar artery.

© 2024 Wei Y et al. *JAMA Network Open.*

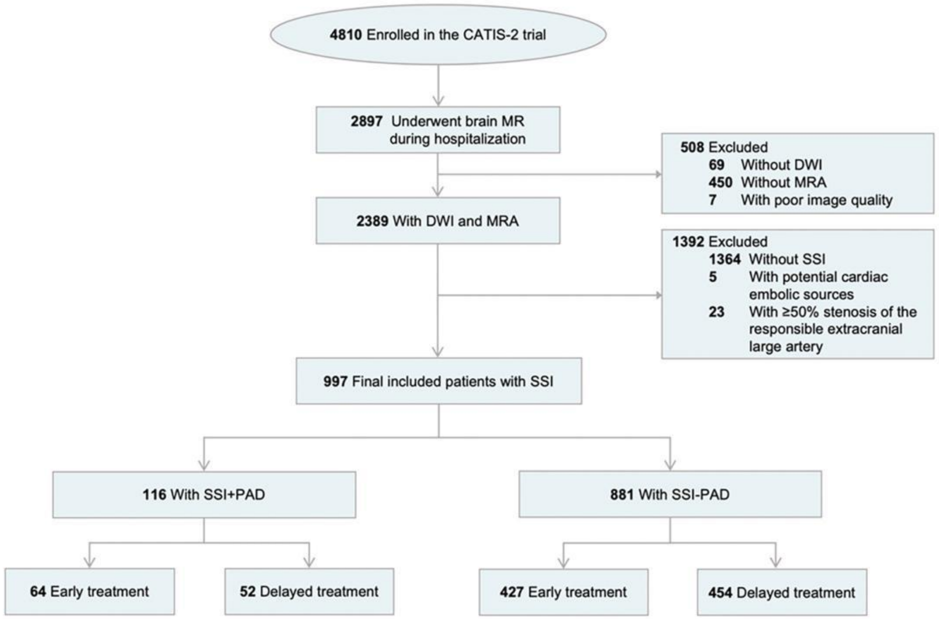

eFigure 2. Flowchart of the Current Study

Abbreviations: MR, magnetic resonance; DWI, diffusion-weighted imaging; MRA, magnetic resonance angiography; SSI, single subcortical infarction; PAD, parent artery disease.

© 2024 Wei Y et al. *JAMA Network Open.*
