# Supplementary material for: Early vs Delayed Antihypertensive Treatment in Acute Single Subcortical Infarction: A Secondary Analysis of the CATIS-2 Randomized Clinical Trial
Source: JAMA Netw Open. 2024 Aug 30;7(8):e2430820. doi: 10.1001/jamanetworkopen.2024.30820 (PMC11365005; doi:10.1001/jamanetworkopen.2024.30820)
Supplement: Supplement 4. — Data Sharing Statement [file jamanetwopen-e2430820-s004.pdf]

Data Sharing Statement

Wei. Early vs Delayed Antihypertensive Treatment in Acute Single Subcortical Infarction. *JAMA Netw Open*. Published August 30, 2024. doi:10.1001/jamanetworkopen.2024.30820

Data
Data available: No
